# Versatile Roles of K63-Linked Ubiquitin Chains in Trafficking

**DOI:** 10.3390/cells3041027

**Published:** 2014-11-12

**Authors:** Zoi Erpapazoglou, Olivier Walker, Rosine Haguenauer-Tsapis

**Affiliations:** 1 Institut Jacques Monod-CNRS, UMR 7592, Université-Paris Diderot, Sorbonne Paris Cité, F-75205 Paris, France; E-Mail: zoi.erpapazoglou@icm-institute.org; 2 Current address: Brain and Spine Institute, CNRS UMR 7225, Inserm, U 1127, UPMC-P6 UMR S 1127, 75013 Paris, France; 3 Institut des Sciences Analytiques, UMR5280, Université de Lyon/Université Lyon 1, 69100 Villeurbanne, France; E-Mail: olivier.walker@univ-lyon1.fr

**Keywords:** Lys63-linked polyubiquitylation, intracellular trafficking, endocytosis, multivesicular bodies, autophagy

## Abstract

Modification by Lys63-linked ubiquitin (UbK63) chains is the second most abundant form of ubiquitylation. In addition to their role in DNA repair or kinase activation, UbK63 chains interfere with multiple steps of intracellular trafficking. UbK63 chains decorate many plasma membrane proteins, providing a signal that is often, but not always, required for their internalization. In yeast, plants, worms and mammals, this same modification appears to be critical for efficient sorting to multivesicular bodies and subsequent lysosomal degradation. UbK63 chains are also one of the modifications involved in various forms of autophagy (mitophagy, xenophagy, or aggrephagy). Here, in the context of trafficking, we report recent structural studies investigating UbK63 chains assembly by various E2/E3 pairs, disassembly by deubiquitylases, and specifically recognition as sorting signals by receptors carrying Ub-binding domains, often acting in tandem. In addition, we address emerging and unanticipated roles of UbK63 chains in various recycling pathways that function by activating nucleators required for actin polymerization, as well as in the transient recruitment of signaling molecules at the plasma or ER membrane. In this review, we describe recent advances that converge to elucidate the mechanisms underlying the wealth of trafficking functions of UbK63 chains.

## 1. Introduction

Ubiquitin (Ub), a highly conserved 76-amino acid polypeptide present in eukaryotes, is conjugated to proteins by the consecutive action of Ub-activating (E1), Ub-conjugating (E2) enzymes, and Ub ligases (E3), which normally link the COOH-terminus of Ub to the ε-amino group of lysine (Lys) residues in target proteins. The ubiquitylation cascade starts with the formation of a thioester bond between Ub and an E1 enzyme, followed by Ub transfer to the active site cysteine of an E2 enzyme, and finally Ub attachment to a substrate with the help of an E3 ligase [1]. Ubiquitylation is a reversible modification, and deubiquitylating enzymes (DUBs) can process Ub precursors or deubiquitylate substrates [2]. Substrates can display monoubiquitylation, multiple monoubiquitylation, or polyubiquitylation, which is the addition of polyUb chains that are interlinked through any one of the seven Lys residues of Ub (K6, 11, 27, 29, 33, 48 and 63) [3]. In addition to the seven internal Lys residues of Ub, the N terminus of Ub also can be used as an attachment point, thereby generating linear Ub chains [4]. This wealth of distinct potential types of ubiquitylation modulates the function, localization, and protein–protein interactions of the tagged substrates through their interactions with multiple partners carrying Ub-binding domains (UBDs). The first described role for ubiquitylation was to target proteins for degradation by the proteasome, a process that most often involves K48-linked Ub (UbK48) chains [1] but also K11-linked Ub chains [5].

Early studies in yeast based on the construction of strains carrying deletions of the genes encoding polyUb (UBI4) and Ub ribosomal protein fusion (UBI1-3) and expressing as sole source of Ub derivatives with unique Ub Lys-to-Arg (KR) substitutions have shown that only UbK48 chains are required for the viability and bulk turnover of short-lived proteins [6]. These precursor studies also showed that yeast cells unable to assemble K63-linked Ub (UbK63) chains display stress resistance [7], deficiencies in DNA repair [6], or impaired ribosomal function [8]. Substrates modified by UbK63 chains represent the second most abundant class of polyubiquitylated proteins, both in yeast [9], and mammalian cells [10]. The known functions of this modification also include mitochondrial inheritance [11], activation of kinases in various signaling pathways [12], and regulation of several steps of the endocytic pathway.

The first protein shown to be modified by (short) UbK63 chains was a yeast plasma membrane transporter, the uracil permease [13]. Since then, an extensive list of plasma membrane proteins have been described to be modified by UbK63 chains in yeast, mammals and plants (Table 1). Whether this specific post-translational modification is required for the internalization of these proteins is a matter of debate. UbK63 chains also have been shown to be involved in a second step of the endocytic pathway: sorting to multivesicular bodies (MVBs) [14,15,16] (for reviews see [17,18,19]). In addition, UbK63 chains have been shown to be required in other steps of the endocytic pathway, or in recycling, and in various forms of autophagy. In the present manuscript, we attempted to include recent available information to shed new and critical light on the functions of UbK63 chains in all of these trafficking events and to present structural data to facilitate an understanding of the assembly, recognition and disassembly of UbK63 chains involved in these processes*.*

## 2. Modification of Plasma Membrane Proteins by UbK63 Chains: Occurrence and Potential Functions

Early studies in yeast led to the discovery of the critical role of ubiquitylation in the internalization of most plasma membrane proteins in this organism [20]. In mammals, the potential role of ubiquitylation of plasma membrane proteins in their internalization was and remains a controversial field, for different reasons. Indeed, the internalization step of endocytosis in mammals can occur through different pathways, and some plasma membrane proteins can be internalized through parallel pathways; ubiquitylation is only one of the possible internalization signals. We will summarize only a few of the emblematic examples illustrating unquestionable cases of the occurrence and role of UbK63 chains in the internalization process, some of the long-standing controversies in this field, or examples of the variety of situations underlying the involvement of UbK63 chains in the internalization process.

### 2.1. E2s/E3s and UbK63 Modification of Endocytic Cargoes

#### 2.1.1. UbK63 Chains and Plasma Membrane Proteins in Yeast

**Figure 1:**
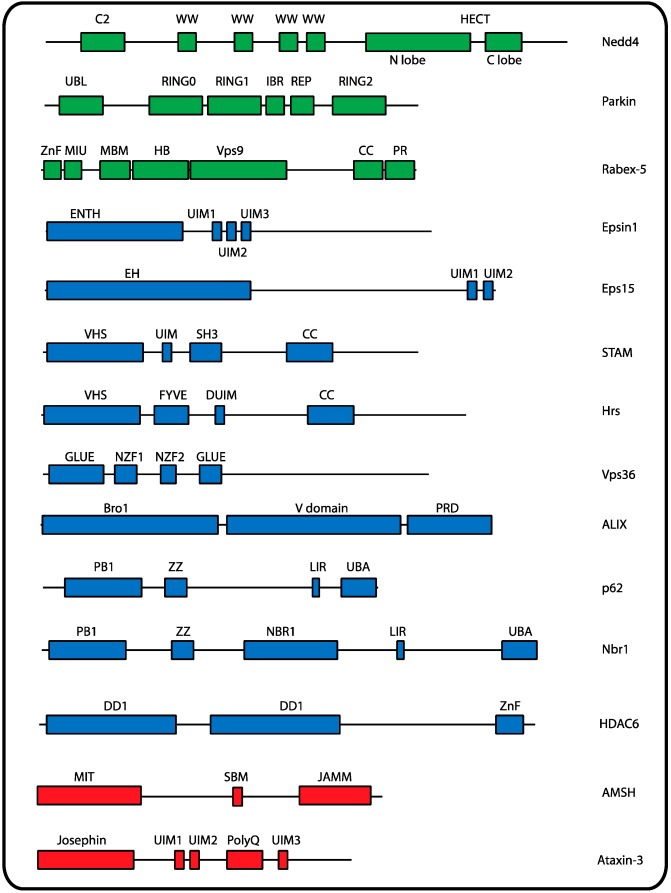
Assembly, disassembly and recognition of UbK63 chains.

**Figure 2:**
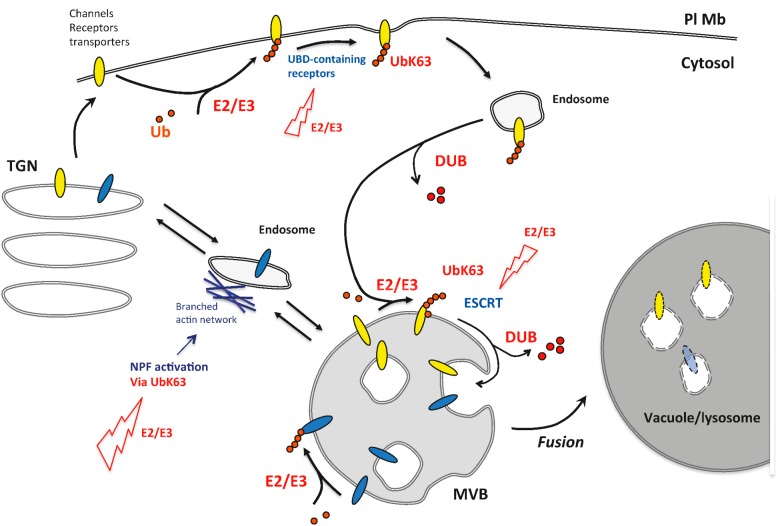
Examples of trafficking steps that involve UbK63 chains.

A preliminary report demonstrated the accumulation of ubiquitylated forms of a plasma membrane protein—the mating pheromone a-factor transporter Ste6—in mutants impaired in the internalization step of endocytosis [21]. Subsequently, it was shown that mutations in the ubiquitylation machinery display an inhibition of the internalization of the α-factor receptor Ste2 [22], the general amino acid permease Gap1, and the uracil permease Fur4 [23]. Rsp5, an unique member of the Nedd4 E3 family in yeast (Figure 1), was identified as the key player in the ubiquitylation and internalization of these three proteins [13,24,25] and subsequently a number of others (reviewed in [17,26] (Table 1). The target Lys residues were identified on some of these proteins, and Lys-to-Arg (KR) mutation of these residues resulted in complete protection against internalization [27,28]. Taken together, these data indicate that ubiquitylation of these proteins acts as a signal triggering internalization of those proteins and their subsequent degradation in the lysosome/vacuole (Figure 2). A question immediately raised by these observations concerns how the described plasma membrane proteins escape degradation by the proteasome. The predominant form of modified Ste2 is monoubiquitylated [22]. On the other hand, the use of a mutant strain with UbK63R as the sole source of Ub revealed that Fur4 undergoes modification of its two close target Lys by short Ub chains that are linked through UbK63 [13]. A very similar ubiquitylation pattern is displayed by Gap1 [28,29]. The occurrence of monoubiquitylation (Ste2) or ubiquitylation by UbK63 chains (Fur4, Gap1, and several other plasma membrane proteins) [13,14,16,29,30] (Table 1) provides an explanation for why all of these proteins escape proteasome recognition.

**Table 1:** List of UbK63 chain targets along different trafficking pathways.

***Plasma Membrane***					
**Organism**	**Target**	**E2/E3**	**DUB**	**Ub Receptor/Adaptor**	**References**
***Yeast***	Uracil permease Fur4	NI ^1^/Rsp5	NI	NI	[13,31]
	General amino acid permease Gap1	NI/Rsp5	NI	NI	[25,29]
	Ferrichrome C transporter Arn1	NI/Rsp5	NI	NI	[30]
	Ferrioxamine B transporter Sit1	NI/Rsp5	NI	NI	[14]
	Lactate transporter Jen1	NI/Rsp5	NI	NI	[16]
***Mammals***	NGF receptor TrkA	UbcH7/TRAF6	CYLD	NI/p62	[32,33]
	MHC class I molecule	UbcH5, Ubc13/K3, K5	NI	Epsin 1, Eps15	[34,35,36]
	Dopamine transporter DAT	UBE2D, UBE2L3/Nedd4-2	NI	Epsin 1, Esp15, Eps15R	[37,38]
	Aquaporin-2 water channel	NI/Nedd4, Cul5	NI	NI	[39]
	Prolactin receptor	NI/SCF^β-TRCP^	NI	NI	[40]
	human κ-opioid receptor	NI	NI	NI	[41]
	LDL receptor	UBE2D/IDOL	NI	NI	[42,43]
	EGF receptor	NI/c-Cbl	NI	Epsin 1, Ankrd 13	[44,45,46]
	Interferon receptor IFNAR1	SCF^β-TRCP^	BRCC36 (BRISC-SHMT2 complex)	NI	[47,48,49]
	α-subunit of Kv11.1 channel hERG-K	NI/CHIP	NI	NI	[50]
	IGF-I Receptor (IGF-IR)	NI/Mdm2	NI	NI	[51]
	TNF Receptor 1 (TNF-R1)	Ubc13/RNF8	NI	NI	[52]
	Cationic amino acid transporter (CAT-1)	NI/Nedd4-1, Nedd4-2	NI	NI	[53]
***Plants***	Auxin carrier PIN2	NI/ RGLG1, RGLG2	NI	TOL proteins	[54,55,56]
***Golgi-Endosomes-MVBs***					
****Organism****	**Target**	**E2/E3**	**DUB**	**Ub Receptor/Adaptor**	**References**
***Yeast***	Rsp5 adapter Sna3	NI/Rsp5	NI	ESCRT ^2^	[57]
	Ferrioxamine B transporter Sit1	NI/Rsp5	NI	,,	[14]
	General amino acid transporter Gap1	NI/Rsp5	NI	,,	[15]
	Carboxypeptidase S Cps1	NI/Rsp5	NI	,,	[15]
	Lactate transporter Jen1	NI/Rsp5	NI	,,	[16]
	Rsp5 adapter Ear1	NI/Rsp5	NI	,,	[58,59]
	Uracil permease Fur4	NI/Rsp5	NI	,,	[60,61]
***Mammals***	Melanocytic protein MART1	NI/Nedd4, Itch	NI		[58,62]
	EGF receptor	NI/NI NI/NI Ubc13/RNF126, Rabring 7 NI/Triad	NI AMSH NI	,,	[63] [64] [65] [66]
	GH Receptor (GHR)	NI/Triad	NI	,,	[66]
	E3 ligase c-Cbl	NI/NI	NI	NI	[63]
	E3 ligase Nedd4-L	NI/NI	NI	NI	[63]
	Solute carrier SLC3A2	NI/NI	NI	NI	[63]
	GTPase H-Ras	NI/Rabex-5	NI	NI	[67,68]
	Actin polymerization regulator WASH	Ube20/MAGE-L2-TRIM27	NI	NI	[69]
	Amyloid Precursor Protein (APP)	NI/NI	NI	NI	[70]
	α-synuclein (wild-type)	NI/Nedd4-1	NI	NI	[71]
***Drosophila***	GTPase H-Ras	NI/Rabex-5	NI	NI	[67]
***Worms***	Caveolin homolog Cav1	Ubc13/NI	NI	NI	[72]
***Viral budding***					
****Organism****	**Target**	**E2/E3**	**DUB**	**Ub receptor**	**References**
***HIV***	Gag	NI/NEDD4-2s	NI	ALIX	[73,74]
***Cytoskeleton***					
****Organism****	**Target**	**E2/E3**	**DUB**	**Ub receptor**	**References**
***Mammals***	Focal adhesion protein paxillin	Ubc13/RNF5	NI	NI	[75]
***Mitochondria***					
****Organism****	**Target**	**E2/E3**	**DUB**	**Ub receptor**	**References**
***Mammals***	Mitofusin Mfn2	UbcH5/MITOL	NI	NI	[76]
	E3 ligase Parkin	NI/NI	NI	NI	[77]
	Ser/Thr kinase PINK1	NI/TRAF6-SARM1	NI	NI	[78]
***Autophagy***					
****Organism****	**Target**	**E2/E3**	**DUB**	**Ub receptor**	**References**
***Mammals***	α-synuclein mutant isoforms pPhosphorylated α-synuclein	NI/Parkin NI/Parkin	NI NI	NI NI	[79] [80]
	DJ-1 mutant isoform (L166P)	Ubc13/Parkin	NI	HDAC6	[81]
	Synphilin-1	NI/Parkin	NI	NI	[82]
	sodium dismutase (SOD1) mutant isoform (G93A)	NI/ΝΙ	Ataxin-3	NI	[83]
	Caspase-8	NI/TRIM13	NI	NI	[84]

^1^ Not Identified; ^2^ For all reported examples, MVB sorting was shown to be dependent on UBD-containing ESCRT complexes.

Schematic representation of the different domains harbored by the proteins involved in trafficking with known connections to UbK63 chains. The sequences are colored according to the following code: E3: green, receptors: blue and DUB: red. Only proteins detailed in the different boxes are present. The definition of the different domains are the following: **C2**: Protein kinase C conserved region 2; **WW**: protein-protein interaction domain containing two conserved tryptophan residues; **HECT**, homologous with E6-associated protein *C*-terminus; **UBL**: Ub like; **RING**: Really interesting new gene; **IBR**: In between ring fingers; **ZnF**: A20-like Zinc finger; **MIU**: Motif interacting with Ub; **HB**: Helical bundle; **Vps9**: Vacuolar sorting protein 9; **PR**: Proline rich; **ENTH**: Epsin *N*-terminal homology; **UIM**: Ub interacting motif; **EH**: Eps15 homology; **VHS**: VPS27, Hrs and STAM; **SH3**: Src homology 3; **CC**: Coiled coil; **FYVE**: Fab1, YGL023, Vps27, and EEA1; **DUIM**: Double UIM; **GLUE**: GRAM-Like Ub-binding in EAP45; **NZF**: Npl4 zinc finger; **BRO1**: BCK1- like resistance to osmotic shock; **PB1**: Phox and Bem1; **ZZ**: ZZ-type zinc finger; **LIR**: LC3 interacting region; **UBA**: Ub-associated; **NBR1**: neighbor of BRCA1 gene 1; **MIT**: Microtubule Interacting and Trafficking; **SBM**: SH3 binding motif; **JAMM**: JAB1/MPN/MOV34; **polyQ**: polyglutamine repeat.

Several channels, transporters and receptors undergo modification by UbK63 chains assembled by various E2/E3 pairs. These endocytic cargoes are recognized for sorting to invaginated regions of the plasma membrane by a number of UBD-containing proteins. Some of these receptors are themselves ubiquitylated. Before/after sorting to early endosomes, ubiquitylated endocytic cargoes may be deubiquitylated. Endocytic cargoes join, at the level of MVBs, proteins being trafficked from the trans Golgi network (TGN). Endocytic cargoes and a subset of membrane-bound proteins trafficked from the TGN are ubiquitylated by UbK63 chains. These MVB ubiquitylated cargoes are recognized by UBD-containing proteins of ESCRT complexes -some of which display a better affinity for UbK63 chains than for monoUb- and sorted to invaginating regions of the MVB. They undergo deubiquitylation by endosomal DUBs in the late step of ESCRT-dependent MVB sorting. Many UBD-containing ESCRT proteins themselves undergo ubiquitylation, some possibly with UbK63 chains. UbK63 chain modification of a NPF (nucleation-promoting factor) also leads to branched actin network at the endosome, required for endosome to TGN retrieval.

The occurrence of a rather general modification of yeast plasma membrane proteins with UbK63 chains is consistent with the predominant mode of UbK63 chain generation by Rsp5 [85,86] due to its structural properties: the N-lobe of the catalytic HECT (Homology to E6AP *C*-terminus) domain contains a non-covalent Ub-binding site that functions to recruit a substrate-conjugated Ub molecule to promote conjugation of the next Ub moiety [87] (Box 2). It must be highlighted that in the latter *in vitro* studies, the UbK63 chains that assemble on Rsp5 substrates are rather long, whereas the modification observed on Gap1 or Fur4 correspond to rather short chains (two-three-Ub long). Whether ubiquitylated forms of Fur4 and Gap1 undergo artifactual or *in vivo* deubiquitylation, or whether molecular mechanisms limit *in vivo* Rsp5-dependent Ub chain extension [27,28], remains to be determined.

Further questions remain concerning the function of these modifications in the internalization process. Fusion of Ub in-frame to a mutant form of Ste2 lacking its cytoplasmic tail Lys has been described to be sufficient to promote its rapid internalization, suggesting that monoubiquitylation could be the prevalent signal for this protein [88]. However, *N*-terminal fusion of Ub to a mutant Fur4 lacking its two target Lys and completely stabilized at the cell surface induces only a low rate of internalization, and fusion of Ub in-frame to wild type Fur4 accelerates the internalization [60]. The use of transporters with mutations in their target Lys and of cells unable to assemble UbK63 chains revealed that two Ub monomers, or a short UbK63 chain, constitute an equally efficient internalization signal for Fur4 [13,27] and Gap1 [15]. Hence, in both cases, the total number of Ub moieties, rather than the UbK63 chains, is the important determinant of the internalization signal. The difference between the efficiency of monoubiquitylation for Ste2 internalization compared to the requirement for additional Ubs for Fur4 or Gap1 may result from the larger hydrophobic core of the two latter proteins, which may require more binding sites to interact properly with the endocytic machinery, or from the presence of a sorting signal on Ste2 that functions redundantly with the Ub-based signal [89]. Indeed, if ubiquitylation is by far the main internalization signal in yeast, there are a few exceptions corresponding to another internalization signal (NPFX) [90] that is present on Ste2 and recognized by the Sla1 protein [89].

#### 2.1.2. UbK63 Chains and Plasma Membrane Proteins in Mammals

Platelet-derived growth factor receptor (PDGFR) and epidermal growth factor receptor (EGFR) were the first mammalian receptors described to be ubiquitylated in a ligand-dependent way [91,92]. These observations were clarified when genetic studies in *Caenorhabditis elegans* revealed that Sli1, the nematode homolog of cCbl, plays a negative regulatory role downstream of Let23, the worm ortholog of EGFR [93], and that cCbl has intrinsic E3 protein ligase activity [94]. Thus, it became clear that the Cbl family of proteins (three isoforms in humans) interact with several receptor tyrosine kinases (RTK) after their ligand-induced activation, leading to their Cbl-dependent ubiquitylation and down-regulation (reviewed in [95]). Given the critical role of RTK-dependent signaling for normal homeostasis and development, aberrant ubiquitylation of these receptors contributes to malignant transformation: both oncogenic and tumor-suppressor forms of Cbl have been reported [96], and mutations in RTK leading to abnormal ubiquitylation are widespread in cancer [97]. Other mammalian plasma membrane proteins have been shown to be ubiquitylated, including channels and transporters. One of the pioneer works in this field was the discovery that the α-subunit of the sodium channel ENaC interacts directly with Nedd4-2 [98], leading to its ubiquitylation and down-regulation [99]. Mutations in ENaC that impair its interaction with Nedd4-2 and its ubiquitylation lead to an inherited hypertension known as Liddle syndrome [100].

Compared to yeast and the predominant role of a unique E3, Rsp5, multiple E3s, both of the HECT and RING (Really Interesting New Gene) subfamilies (Figure 1) were shown to be involved in the ubiquitylation of mammalian plasma membrane proteins (recently reviewed in [18]). In addition, some proteins can be substrates of different E3s [32,101]. Characterization of the type of modification of mammalian plasma membrane proteins was also initially more challenging than in yeast: complete elimination of the expression of endogenous Ub and its replacement by mutant Ub was only recently achieved [102]. Thus, a clear demonstration of the presence of plasma membrane proteins modified by UbK63 chains was mainly obtained using siRNA of the E2 assembling specifically UbK63 chains, by sensitive and quantitative mass spectrometry methods, or using the recently available antibodies specific for UbK48 or UbK63 chains [103]. It is now clear that an expanding list of mammalian membrane proteins are modified by UbK63 chains at the plasma membrane (Table S1). However, it is not always clear whether ubiquitylation is involved in internalization or in downstream events in the endocytic pathway.

##### Emblematic Examples of E2/E3 Pairs that Lead to Modification of Their Target Endocytic Cargoes by UbK63 Chains

The first and possibly the most completely documented example of the modification of a plasma membrane protein by UbK63 chains for internalization is the Major Histocompatibility Complex type I (MHC I). This protein was shown to be ubiquitylated by the Ub ligase K3 (also known as MIR1) encoded by the Kaposi virus, a modification that is essential for its down-regulation [34,104]. K3 was shown to interact with UbcH5 and Ubc13 [35]. The latter E2, in complex with Mms2, is the unique E2 known to specifically promote the formation of UbK63 chains [105] (Box 1). siRNA-mediated depletion of Ubc13 leads to the accumulation of a monoubiquitylated form of MHC I, resulting from prior ubiquitylation by the UbcH5/K3 pair. Depletion of UbcH5 together with Ubc13 completely prevents MHC I ubiquitylation and internalization, as determined using a cytofluorometric-based assay. Overexpression of UbK63R leads to the accumulation of monoubiquitylated MHC I and stabilizes it at the cell surface. Taken together, these data demonstrate that MHC I undergoes monoubiquitylation by the UbcH5/K3 pair, followed by UbK63-linked ubiquitylation by the Ubc13/K3 complex. These modifications are required for efficient internalization [35].

**Box 1. Formation of UbK63 Chains by the Mms2-Ubc13 Complex**

Active E2s possess a core Ub-conjugating (UBC) domain, which contains the catalytic Cys residue and interacts with E1s. Ub conjugating enzyme variant (UEV) proteins also have a UBC domain but lack an active site Cys residue [106]. For UbK63 chains, a heterodimer formed by an E2 protein (Ubc13), which harbors a catalytic cysteine residue and a UEV (Mms2) [105] are required for polyubiquitin (polyUb) chain production [107]. Several structures or models of the Mms2/Ubc13 non-covalent complex have been reported (see Supplementary Table S2 and Figure IA) [107,108,109,110], all with almost identical conformation. This complex is characterized by a narrow interface of ~30 Å long and 10 Å wide, burying a total of ~1500 Å^2^ of solvent-accessible surface area [110], with a tight dissociation constant of 49 ± 7 nM (Supplementary Table S1) [109]. In addition to the catalytic hUbc13-Cys87 residue, the hUbc13-Asp81 residue has been shown to be essential for the synthesis of UbK63 diUb chains and it has been suggested that this residue positions the Ub-Lys63 residue within the active site [107].

**Figure I:**
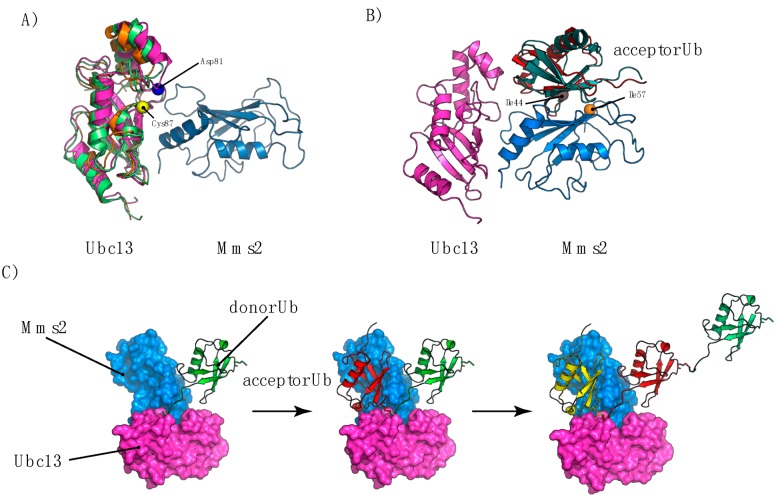
Superimposition of several structure of the Mms2/Ubc13 complex (**A**), aligned on the Mms2 structure (PDB codes: 2GMI: pink, 1JAT: green, 1J7D: orange). (**B**) Structure of the Mms2/Ubc13/Ub^acceptor^ complex (1ZGU: red, 2GMI: green). (**C**) Model of Lys63-linked polyUb chain formation based on the PDB code 2GMI, with a Ub donor molecule delivered to the Mms2/Ubc13 complex (green) by E1. An acceptor Ub then binds to the acceptor binding site of the Mms2/Ubc13 complex, allowing Lys63 to form an isopeptide bond with the donor Ub. The previous donor Ub (green) then becomes the acceptor Ub, and a new donor Ub is loaded by another E1.

UbK63 chain assembly is dependent on the unique structural organization of the UEV-Ubc13 family of heterodimeric enzymes, promoting the discharge of a covalently bound donor Ub to a lysine residue of the acceptor Ub. The non-covalent binding of an acceptor Ub to hMms2 has a dissociation constant of 98 ± 5 µM, or 28 ± 6 µM in the presence of Ubc13 [109]. The Ub/Mms2 interface is centered on Ub-Ile44 and Mms2-Ile57 (Ile67 in humans), and the mutation of either of these two residues causes defective polyUb chain formation [111]. Eddins and coworkers solved the structure of a Mms2/Ubc13 complex covalently bound to Ub, thereby improving our understanding of Lys63-linked polyUb chain assembly [108]. In the crystal, the *C*-terminus of a donor Ub is covalently bound within the active site, whereas the acceptor Ub is involved in a non-covalent interaction with the Mms2 of a neighboring complex (Figure IB). This is consistent with the NMR structure of the Mms2/Ub complex [112] and suggests a mechanism in which Mms2 positions the acceptor Ub to ensure Lys63 linkage specificity and isopeptide bond formation. After the transfer of the donor Ub from E1 to the active site of E2, the acceptor Ub binds the Mms2/Ubc13 interface before isopeptide bond formation (Figure IC). In this configuration, the Lys48 residue of the acceptor Ub is 22 Å away from the Ubc13 active site, preventing the formation of the Mms2/Ubc13 complex required for UbK48 chain formation.

One of the most studied examples of a receptor undergoing Ub-mediated down-regulation is most likely that of RTK EGFR, which controls signaling pathways involved in proliferation, migration, survival, and adhesion [113]. It is also a field of investigation characterized by multiple controversies. The initial attempt to determine the type of ubiquitylation of EGFR was conducted using antibodies that were described to specifically recognize either polyubiquitylated proteins (FK1 plus FK2) or monoubiquitylated and polyubiquitylated proteins (P4D1). Immunoprecipitation of EGFR after ligand-stimulation followed by immunoblotting with P4D1 revealed an intense signal in the appropriate high molecular weight range, whereas only a very weak signal was detected with FK1 plus FK2 [114]. The authors concluded that EGFR was mainly monoubiquitylated at multiple sites, and the same was observed for PDGFR. They then investigated the role of monoubiquitylation by following the fate of a chimeric protein consisting of EGFR extracellular and transmembrane domains fused in-frame to a mutant Ub that could not be further conjugated. The latter protein was internalized at half the rate of wild type EGFR. The authors concluded that monoubiquitylation was sufficient for EGFR internalization [114], an interpretation that has long been considered the current paradigm.

A few years later, EGFR ubiquitylation following ligand stimulation was followed using sensitive and quantitative (Ub-AQUA technique) mass spectrometry. The data obtained revealed unambiguously that EGF treatment induces ubiquitylation of EGFR at several Lys residues, with more than 50% in the form of polyUb chains, primarily short UbK63 chains (2–3-residues long), as well as a minor fraction in the form of UbK48 chains (Table 1) [44]. This result most likely indicates that FK1 and FK2 antibodies do not efficiently recognize UbK63 chains. Furthermore, this observation raises questions regarding the structural explanation for why Cbl assembles mostly UbK63 chains on EGFR. Extensive analysis of the fate of mutant EGFR lacking an increasing number of target Lys residues, which progressively impairs EGFR ubiquitylation, suggested that if receptor ubiquitylation can mediate internalization in a process that requires Cbl and clathrin, the receptor can also be internalized by multiple additional mechanisms. According to these authors, EGFR ubiquitylation, and notably its modification by UbK63 chains would be mainly involved in later steps of EGFR endocytosis [64,115]. However, others have shown that *C*-terminal fusion of four Ubs connected linearly (a tag with some structural analogy with four Ubs linked through UbK63) promoted constitutive clathrin-dependent internalization of EGFR, bypassing the requirement for ligand-induced activation, the tyrosine kinase activity of the receptor, and the involvement of Cbl [116]. Thus, EGFR ubiquitylation can indeed promote its internalization.

The dopamine (DA) transporter (DAT) expressed in dopaminergic neurons is a good example to demonstrate the power of all of the available biochemical tools to characterize the type of ubiquitylation of a plasma membrane protein. Both in humans and rat neuronal cells, DAT undergoes basal and massive ubiquitylation following activation of protein kinase C (PKC) by phorbol ester (phorbol 12-myristate 13-acetate, PMA) [117]. PMA-induced DAT ubiquitylation depends on Nedd4-2 [37]. Analysis by mass spectrometry of purified DAT following PMA treatment and the use of an antibody specific for UbK63 chains permitted the identification of some of the target Lys and demonstrated that this transporter is mostly modified by UbK63 chains (Table 1) and trace amounts of UbK48 [117]. This finding is in agreement with the preferred assembly of UbK63 chains by Nedd4 [118] (Box 2), as described for Rsp5 [87]. Interestingly, based on the molecular weight of either ubiquitylated wild type DAT or variant DAT mutated in some of the target Lys, it appears that each ubiquitylated DAT carries four Ub moieties linked by UbK63 on one of its target Lys [38]. This again raises questions regarding the mechanism(s) underlying the limitation in the number of added Ub moieties. The fate of wild type DAT, or multilysine mutant DAT, was followed using parallel approaches combining cell surface biotinylation, immunofluorescence and antigen-driven internalization assays performed in the presence or absence of inhibitors of potential recycling. The data obtained demonstrated convincingly that DAT ubiquitylation is required for its internalization [119].

**Box 2. Formation of UbK63 Chains by a HECT-E3**

The Mms2/Ubc13 heterodimer specifically assembles UbK63 chains, but an E3 ligase is required for the assembly of these chains on a protein acceptor. There are three classes of E3 ligases [120]. The really interesting new gene (RING) family enzymes catalyze the direct transfer of Ub from the E2 enzyme to the substrate, simultaneously binding both the E2~Ub thioester and the substrate [121,122]. By contrast, the homology to E6AP C terminus (HECT) and the RING-between-RING (RBR) family E3s ubiquitylate substrates in a two-step reaction in which Ub is transferred from the E2 to an active-site cysteine residue in the E3 and then from the E3 to the substrate [123,124]. The mechanistic role of the E3 ligase is to bind the E2-Ub thioester and the acceptor protein for further transfer of the Ub from the active-site cysteine of the E2 to the substrate lysine residue or another Ub moiety for chain elongation. Despite numerous structural studies of E2-Ub and E3 complexes, our understanding of the mechanism by which Ub is transferred to the substrate or chains are elongated remains limited and mostly hypothetical.

The enzymes of the HECT-domain E3 family are involved in cell trafficking via Nedd4 or yeast Rsp5 E3 ligases. HECT-domain E3 ligases operate through a two-step mechanism [125]. Ub is first transferred from the E2 active cysteine to a cysteine of the HECT domain and, thus, to a lysine substrate. Several structural studies have increased our knowledge of Ub substrate transfer or polyUb chain elongation [87,112,126,127]. The HECT domain contains two substructures, a N lobe essential for the transfer of Ub from E2 to E3 [128] and a C lobe containing the catalytic cysteine residue essential for enzyme processivity [87,127]. The C lobe is free to rotate around a flexible hinge that tethers it to the N lobe [87]. UbK63 chain assembly is achieved through the sequential addition of Ub molecules from the catalytic cysteine residue to the distal lysine residue of the growing chain [127,129]. However, the precise mechanisms involved remain unclear due to the lack of structural data. Superimposing two recently determined structures, for a HECT(Nedd4)-Ub complex [112] and a UbcH5B-Ub/HECT(Nedd4L) complex [128], revealed a possible mechanism for the transfer of Ub from E2 to E3 (Figure IA–C), in which the interaction of E2 with the N lobe facilitates the transfer of the thioester bond from the E2 to the E3, on which the donor Ub has already been transferred to the C lobe ready for transfer to the substrate. The HECT(Nedd4)-Ub structure contains an additional Ub that is non-covalently bonded to the N lobe and could serve as a potential substrate for UbK63 chains (Figure IB). This raises questions about how Ub can be transferred from the E3 to the substrate. The structure of the ternary HECT(Rsp5)-Ub/Sna3 complex showing the yeast Rsp5 simultaneously cross-linked to Ub and Sna3 [126], provides one possible answer. In this structure, the transfer of Ub from the E3 to the substrate requires a 130° rotation of the HECT domain C lobe, still covalently bound to Ub. This structural organization would reduce conformational flexibility and move the E3-Ub intermediate close to the substrate for the ligation reaction (Figure IC) [130]. Furthermore, the substrate exposes its PPXY motif for a non-covalent interaction with the Rsp5'WW3 domain during Ub attachment [131,132,133]. Overall, this indicates that the HECT E3 prioritizes potential substrate lysines according to their orientation with respect to a catalytic center for ubiquitylation.

**Figure I:**
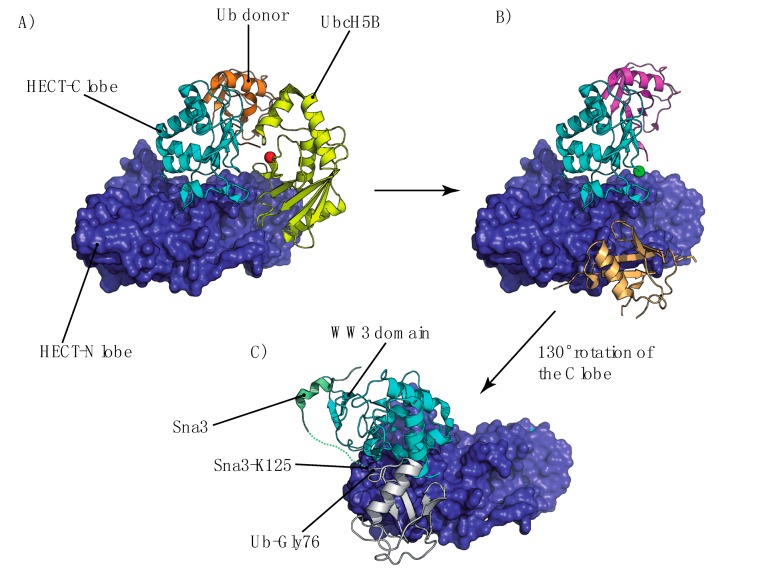
Model of substrate ubiquitylation or polyubiquitylation by UbK63 chains by enzymes of the HECT-domain E3 family. (**A**) The HECT N lobe binds the charged UbcH5B-Ub (active cysteine represented by a red sphere) thioester (PDB code 3JW0) and transfers Ub (**B**) to the catalytic cysteine (green sphere) in the C lobe (PDB code 4BBN). (**C**) The C lobe-Ub thioester then rotates 130° to bring the donor Ub to the substrate-binding site, where it can be ligated to an acceptor lysine in the substrate (PDB code 4LCD).

Parkin belongs to the RBR-domain E3 family and has features in common with both HECT and RING E3s [125]. Following its phosphorylation by PINK1, Parkin induces polyubiquitylation (Lys48-, Lys63, Lys27-linked), leading to the recruitment of Ub-binding adaptor protein p62 [134]. Parkin can also promote the degradation of misfolded proteins through Lys63-linked ubiquitylation, marking these proteins for clearance by autophagy via the adaptor protein HDAC6 [135]. Parkin contains a catalytic cysteine residue in RING2 (Figure 1) that mediates ubiquitylation in a HECT-like mechanism [124] whereas RING1 plays the canonical role of recruiting the loaded E2 [136].

Finally, the interferon (IFN)-α/β receptor IFNAR1, which plays a role in antiviral defense, undergoes ligand-induced phosphorylation within its *C*-terminal tail. This enables its polyubiquitylation, mediated by SCF^βTrcp^ (Skp Cullin F-box) [47], a multisubunit RING E3 ligase that is usually known for targeting its substrates to the proteasome [1]. Mass spectrometry analysis of IFNAR1 revealed that after IFN treatment, this receptor is modified by both UbK48 and UbK63 chains (Table 1), raising questions about the mechanisms underlying the generation of both types of Ub chains by this E3. Overexpression of various UbKR mutants indicated that both type of chains are necessary but not sufficient for the rapid internalization of the receptor [47].

##### The Unexpected Role of CHIP1/Ubc13 in the Internalization of the Growth Hormone Receptor GHR

The growth hormone receptor GHR, a prototype cytokine receptor, undergoes ligand-induced endocytosis and is one historical example of a receptor that was shown to be ubiquitylated and revealed the association between Ub and endocytosis: Chinese hamster ovary cells deficient in the E1 enzyme displayed deficient endocytosis of this receptor [137]. The link between ubiquitylation and GHR endocytosis was found to be extremely complex. Although the endocytosis of GHR depends on the binding and activity of the E3 SCF^β^^TrCP^, ubiquitylation of the receptor itself is not required for its internalization [138]. Intriguingly, in addition to SCF^β^^TrCP^, another E3 ligase, CHIP (*C*-terminus of Hsc70-interacting protein), was shown to be required for proper GHR internalization. CHIP is a U-box-containing E3 that acts with two E2s, either UbcH5a or Ubc13, to mediate the modification of its substrates by UbK48 or UbK63 chains, respectively. siRNA of CHIP or Ubc13 was shown to decrease the rate of GHR internalization and consequently increase cell-surface amounts of GHR. It does not affect other endocytic cargoes such as transferrin receptor or EGFR. CHIP interacts directly with GHR at a site that partially overlaps with the site of interaction between GHR and βTrCP, but it does not appear to ubiquitylate GHR. It acts on GHR internalization after SCF^β^^TrCP^ but prior to clathrin-mediated GHR endocytosis [139]. The target of CHIP/Ubc13 important for GHR endocytosis remains to be identified. It should be underlined that CHIP also plays a role in quality control processes via the ubiquitylation of mutant misfolded plasma membrane proteins [50] (Table 1).

#### 2.1.3. UbK63 Chains and Plasma Membrane Proteins in Plants

In plants, the role of Ub in the endocytic pathway has emerged over the last few years. One of the first reported examples of an ubiquitylated endocytic cargo was that of the high-affinity iron transporter IRT1. IRT1, a major player in iron homeostasis in plants, is highly expressed in iron-starved root peripheral cells. It was shown to be mainly located in the trans Golgi network/early endosomes as a result of constitutive endocytosis and recycling. IRT1 is ubiquitylated, and based on its detection with P4D1 antibody and lack thereof with FK1 antibody, it has been proposed to be monoubiquitylated [140]. Given the present known limitations of the FK1 antibody, the state of ubiquitylation of IRT1 awaits re-evaluation. Mutation of two target Lys strongly decreased IRT1 ubiquitylation and stabilized the transporter at the plasma membrane, suggesting either impairment in internalization or increased recycling as a consequence of inhibition of endosome-to-vacuole trafficking [140].

To date, to the best of our knowledge, the unique documented example of a plant plasma membrane protein modified by UbK63 chains is the auxin carrier transporter PIN2 of *Arabidopsis Thaliana* (Table 1). PIN2 is one of the exporters of the phytohormone auxin, and thus, it participates in numerous aspects of plant development, notably root gravitropism. PIN2 is modified by UbK63 chains, as revealed by its immunodetection with an antibody against UbK63 chains, and auxin treatment increases this ubiquitylation. Ubiquitylation of PIN2 was shown to be decreased in *rlg1 rlg2* mutants, deficient in the E3 ligases that together with Ubc13 are known to mediate the formation of UbK63 chains *in vitro*. Mutation of multiple Lys in PIN2 (pin2^KR^) stabilizes the exporter. Its internalization, but not its degradation, can be triggered by the in-frame fusion of a single Ub to the pin2^KR^ mutant, suggesting the involvement of UbK63 chains at later steps of the endocytic pathway [54].

### 2.2. Potential Receptors of Ubiquitylated Endocytic Cargoes

Ub receptors in the endocytic machinery were identified in parallel in yeast and in mammals. A hydrophobic patch at the surface of Ub (around Ub I44) is known to interact with many UBDs [141]. In contrast to wild type Ub, UbI44A fused in-frame at the *N*-terminus of a truncated form of Ste2 lacking its target Lys was unable to restore Ste2 internalization, suggesting that UBD-containing receptors play a critical role in the internalization of ubiquitylated yeast plasma membrane proteins [142]. Indeed, several proteins carrying various UBDs have been shown to play a role in the internalization step of endocytosis. Such is the case for Ede1, the yeast equivalent of mammalian Eps15 [143]. Ede1 carries a UBA (Ub-associated) domain, which was identified in precursor informatics studies as a domain that is present in a subset of enzymes in the Ub pathway [144]. The proteins Ent1 and Ent2 [145], yeast homologs of epsin (Figure 1), carry UIM (Ub-interacting Motif) domains, and indeed, they and the Ede1 UBA domain bind to Ub [146,147]. They also play a critical role in the internalization process [145]. These three proteins, which also bind membranes, have been proposed to act as receptors of ubiquitylated endocytic cargoes [146]. However, cells lacking Ede1 UBA and Ent1/2 UIM still internalize ubiquitylated Ste2 [148]. The yeast amphiphysin-like protein Rvs167 and the protein Sla1 are both involved in the internalization step of endocytosis [149,150] and interact with Ub [151]. Therefore, they could potentially play a role as adapters, at least in the absence of Ede1 and Ent1/2 UBDs. However, with the present knowledge of clathrin-mediated endocytosis in yeast characterized by a successive recruitment of these proteins (and many others) at sites of endocytosis in a process involving actin polymerization, it can be expected that receptors of ubiquitylated proteins would be recruited at early time points, which is only the case for Ede1 [152]. Hence, the molecular mechanisms underlying the recognition and subsequent internalization of ubiquitylated endocytic cargoes in yeast remain to be defined.

Many mammalian ubiquitylated endocytic cargoes have been shown to undergo clathrin-mediated endocytosis [35,37,45,48]. Since the first description of this internalization route, the involvement of adaptors in the recognition of endocytic cargoes was postulated because clathrin does not bind cargoes or membranes [153]. These adaptors were expected to display three properties: binding to clathrin, membranes, and endocytic signals in cargoes. Epsins have all of these properties in the case of ubiquitylated endocytic cargoes: they carry an *N*-terminal epsin homology domain (ENTH) that binds phosphoinositides, followed by two UIM and an unstructured region that contains motifs for clathrin binding (reviewed in [154]). Indeed, the siRNA of epsin and of the epsin-partner, the UIM-containing protein Eps15, were shown to impair K3-dependent internalization of MHC I [35], as well as internalization of DAT [37] or EGFR [45]. Moreover, following PMA treatment, ubiquitylated DAT was shown to interact directly or indirectly with epsin and Eps15 [37]. Similarly, after ligand stimulation, ubiquitylated EGFR was shown to interact with epsin in an UIM-dependent way [45]. More precisely, siRNA-mediated epsin depletion was shown to inhibit EGFR translocation to the central region of clathrin-coated pits [45].

The above examples suggest a model in which a signal (ligand, PMA) triggers cell-surface ubiquitylation by UbK63 chains, which are able to promote interactions with UBD-containing receptors (notably epsin) and the transfer of endocytic cargoes to the interior region of clathrin-coated pits (Figure 2). In agreement with a role for UbK63 chains in the internalization of MHC I, DAT or EGFR, epsin 1 was shown to bind preferentially to UbK63 chains with its two UIMs in tandem, separated by a convenient inter-UIM region [155] (Figure 1 and Box 3). The involvement of epsin as a receptor of ubiquitylated cargoes is not limited to its role in clathrin-mediated endocytosis. EGFR has been proposed to undergo Ub-mediated clathrin-independent endocytosis followed by receptor degradation at a high EGF concentration. In this case, epsin was shown to be required for receptor internalization via a raft-dependent pathway [156].

**Box 3. Detection of UbK63 Chains as an Internalization Signal**

Once attached to the target protein, the Ub or polyUb tag must be decoded and interpreted. This is the task of the Ub-binding domains (UBDs), modular domains present in diverse cellular proteins [141]. One of the signals to be decoded is that of the UbK63 chains discussed throughout this review, including Lys63-Ub_2_. Unlike Lys48-Ub_2_ chains, which are dynamic in solution and adopt a compact structure in the closed state [157,158], Lys63-Ub_2_ chains adopt an extended conformation in solution [159,160,161,162]. Epsin and Eps15 are endocytic adaptor proteins that can link Ub cargoes to the clathrin-mediated endocytic machinery [163]. They interact with each other and contain UIM domains [164] (Figure 1). Epsin favors binding to Lys63-linked polyUb, which is consistent with the requirement of multiple Ub moieties as an internalization signal [165].

**Figure I:**
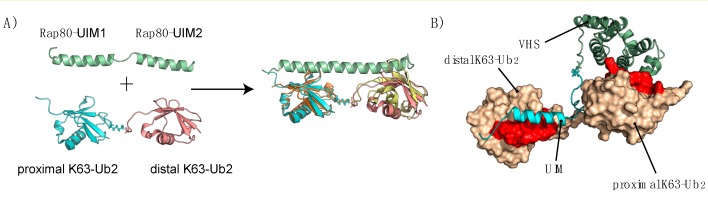
(**A**) Structure of the Rap80^UIM1-UIM2^/K63-Ub_2_ complex. The UIM1 and UIM2 domains are connected by a flexible linker (left), which adopts a helical structure upon binding to K63-Ub_2_. The NMR structure of the complex (PDB code 2RR9, pink and K63-Ub_2_ cyan) and the X-ray structure (PDB code 3A1Q, yellow and K63-Ub_2_ orange) are aligned with respect to the Rap80 structure. (**B**) Model of the structure of the STAM2^VHS-UIM^/Lys63-Ub_2_ complex obtained by NMR and showing the strong specificity of the VHS and UIM domains for the proximal and distal Ub, respectively.

No structural information has yet been obtained for the Epsin/Lys63-Ub_2_ or Eps15/Lys63-Ub_2_ complexes but a hypothetical structural organization can be proposed on the basis of homologous systems. Indeed, Epsin and Eps15 both contain multiple UIM (Ub interacting motif) domains as do many other proteins (S5a, Vps27, Rap80, Ataxin-3 for instance) but they differ in the length of their intervening linker. The inter-domain linker connecting UIM2 and UIM3 in Epsin1 is of similar length to that of Rap80 and binds specifically to Lys63-Ub_2_, whereas the UIM1-UIM2 linker binds neither Lys63-Ub_2_ nor Lys48-Ub_2_. This specific behavior can be attributed to the length of the UIM2-UIM3 linker, and its sequence [155,166]. The linker connecting the Rap80-UIM1 and Rap80-UIM2 domains becomes structured upon binding to Lys63-Ub_2_ [166] and uses mono- and multivalent interactions with polyUb chains to achieve enhanced affinity [167] (Figure IA).

If epsin is probably the common receptor involved in recognition of ubiquitylated endocytic cargoes modified by UbK63 chains, variations of this model have been reported. Interestingly, IFNARI ubiquitylation (mostly with UbK63 chains) appears to promote a conformational change in the receptor, leading to the exposure of a linear endocytic motif that interacts with the AP2 endocytic adapter [48], the first identified adaptor in clathrin-mediated endocytosis.

The role of ubiquitylation events in the internalization step of endocytosis is not limited to the modification of endocytic cargoes. Precursor studies have shown that some UBD-containing proteins, notably Eps15 and epsin, are themselves ubiquitylated [168,169]. These proteins were initially described to be monoubiquitylated, a process that depends on the UIM domain [168,169]. This process, characterized as “coupled monoubiquitylation” (reviewed in [170]) is based on the interaction between an UBD and an E3-loaded Ub, triggering the ubiquitylation of the former on a target Lys outside the UBD [171]. This modification has been proposed to lead to an intermolecular interaction between the UBD and the Ub on the target Lys and thus would abolish the ability of UBD to bind ubiquitylated cargoes [172]. However, *in vivo* experimental data are still required to support this hypothesis.

More generally, many proteins of the yeast endocytic machinery, some carrying an UBD, were identified in proteomic studies as being ubiquitylated [9]. Some of these proteins were shown to be ubiquitylated by Rsp5 [148,151]. In addition to its role in the ubiquitylation of endocytic cargoes, this E3 was described to be required for fluid-phase endocytosis [24], organization of the actin cytoskeleton, and *in vitro* polymerization of actin [173]. In agreement with a role for ubiquitylation/deubiquitylation events in the functions of the endocytic machinery, DUB Ubp7 was recently shown to arrive at yeast endocytic sites at late time points. Moreover, the simultaneous deletion of *UBP7* and *UBP2* resulted in elongation of endocytic coat protein lifetimes [174]. Among the potential DUB targets, purified ubiquitylated Ede1, which undergoes Rsp5-dependent ubiquitylation [148], was shown to be deubiquitylated *in vitro* by Ubp7 and Ubp2 acting simultaneously [174]. Given the preference of Rsp5 and Ubp2 for assembly and disassembly of UbK63 chains, respectively [86], Ede1 could potentially be modified by UbK63 chains. The role of this potential modification remains to be defined. More generally, the molecular mechanisms linking ubiquitylation/deubiquitylation events to cytoskeleton organization at endocytic sites and whether UbK63 chains play a role in these processes remain open questions.

### 2.3. DUBs with UbK63 Specificity and Their Role at the Plasma Membrane

If ubiquitylation has been described in the case of so many plasma membrane proteins, the numbers of ubiquitylated receptors/transporters/channels at each time point are very low, even when their ubiquitylation is required for their internalization. Hence, it is likely that DUBs might be involved in the regulation of the ubiquitylation state of these proteins (Figure 2). Surprisingly, little is known about the involvement of DUBs in this field.

DUBs (more than 100 in humans) are divided into several families (Box 5). Four are cysteine proteases, and there is one family of metalloproteases. Many DUBs are in a conformation that requires substrate binding or an association with partners to be active. Indeed, most DUBs are modular; in addition to catalytic domains, they often display UBD and various protein–protein interaction domains that are involved in direct substrate binding, interactions with scaffolds, or in DUB localization. Many DUBs are also recovered from complexes with E3s [175,176]. The specificity of some DUBs for UbK63 chains could be achieved by preferential binding through recognition of a substrate near the K63-G76 isopeptide bound, or by the position of the polyUb chain within the enzyme catalytic site [175,177,178,179,180] (see also Box 5).

A historical example of a receptor that is modified by UbK63 chains is NGF (nerve growth factor) receptor TrkA. Upon NGF binding, the E3 TRAF6 (tumor necrosis factor (TNF) receptor-associated factor 6) triggers TrkA ubiquitylation with UbK63 chains and subsequent internalization [181]. This modification is also dependent on the recruitment to TRAF6 of the multifunctional adapter p62/sequestosome [181]. Interestingly, p62 also acts as a scaffold between TRAF6 and the DUB CYLD (cylindromatosis tumor suppressor) [33], which disassembles UbK63 chains from target proteins [179]. Thus, p62 has a bifunctional role, ensuring a fine-tuned regulation of TrkA K63-linked ubiquitylation.

A tripartite complex involving a plasma membrane substrate, an E3 and a DUB also has been described in the case of the sodium channel ENaC. The DUB Usp2-45 was shown to interact with both ENaC and Nedd4-2 and to counteract Nedd4-2-mediated ENaC ubiquitylation and down-regulation of ENaC [182]. However, no information has been reported concerning the type of chain specificity of this DUB, although it is reasonable to assume a specificity towards UbK63 chains, because of the type of modifications of a number of Nedd4-2 substrates [118].

Another striking example of a DUB that is involved in the regulation of receptor endocytosis was reported in the case of IFNAR1 [49]. Pulldown experiments revealed the interaction between activated IFNAR1 and SHMT2 (serine hydroxymethyltransferase 2). The latter protein was identified in large-scale studies as a partner of Brcc36, a subunit of the deubiquitylating complex BRISC (BRCC36 isopeptidase complex) belonging to the JAMM family of DUBs [177]. BRISC specifically disassembles UbK63 chains *via* its catalytic subunit Brcc36. The resulting BRISC/SHMT2 complex deubiquitylates UbK63-modified forms of ubiquitylated IFNAR1, thus counteracting its internalization and degradation and regulating IFNAR1 signaling [49]. *In vitro* studies have provided some insights on the mechanism of action of Brcc36. The specificity for endocleavage of UbK63 chains by Brrc36 [178] was shown to result from the orientation of the Lys63-linked isopeptide bound relative to the Bbrc36 active site [177].

## 3. UbK63 Chains and Sorting to Multivesicular Bodies

During their transfer to degradative compartments, lysosomes (mammals) or vacuoles (yeast and plants), ubiquitylated proteins delivered from the plasma membrane or the Golgi apparatus are sorted to multivesicular bodies (MVBs). MVBs were first described in pioneer ultrastructural studies in mammals [183]. They are unique compartments in that the limiting membrane of the organelle buds towards the lumen to generate internal lumenal vesicles (ILVs), to which specific proteins are sorted. When MVBs fuse with the lysosome/vacuole, the internal vesicles and their cargoes are degraded by proteases and lipases, whereas proteins that remain at the MVB-limiting membrane are recovered at the lysosomal/vacuolar membrane.

MVBs have been observed by electron microscopy far more recently in yeast than in mammals [184]. The machinery involved in MVB biogenesis, the so-called ESCRT complexes (Endosomal Sorting Complexes Required for Transport), is conserved from yeast to humans. ESCRT complexes were identified, by genetic studies in yeast (reviewed in [185]) (It must be noted that an MVB sorting defect is easily visualized in yeast, where the vacuole is the biggest organelle: in cells deficient in MVB sorting, GFP-tagged MVB cargoes are recovered at the vacuolar membrane instead of the vacuolar lumen.). Simultaneous studies by several groups established that ubiquitylation of MVB cargoes is required for their sorting to ILVs: biosynthetic membrane proteins destined for the vacuolar lumen are ubiquitylated. Mutation of target lysine(s) results in mis-sorting and accumulation at the vacuolar membrane. Normal sorting to ILVs is rescued by the fusion of a single Ub molecule at the amino terminus of the mutant proteins. In addition, Ub fused in-frame to proteins destined for the vacuolar membrane can divert them to the MVB and subsequently the vacuolar lumen [186,187,188]. Consistent with this Ub-dependent MVB sorting, several proteins of the ESCRT 0, I and II were shown to contain various UBDs (reviewed in [185]). A model was proposed according to which the ubiquitylated cargoes would be transferred sequentially from ESCRT 0 to ESCRT III in parallel with the progressive deformation of the endosomal membrane [189] prior to the final scission of ILVs and release of the Ub moiety, a process mediated in yeast by the DUB Doa4 [190] (Figure 2). Impaired MVB sorting leads to plasma membrane recycling of endocytic cargoes and thus to major signaling defects. Indeed, mutations in several of the ESCRT proteins results in a variety of diseases including cancers (reviewed in [170,191]).

The observation that a single Ub fused in-frame to mutant lysine-less MVB cargoes can direct them to MVBs initially supported the notion that monoUb is the prevalent signal for MVB sorting. Thus, it was unexpected that a DUB specific for the disassembly of UbK63 chains, AMSH [associated molecule with the Src homology 3 domain of signal transducing adaptor molecule (STAM)], interacted with several ESCRT proteins in mammals and plants and participated in the sorting process [192,193]. Below, we present observations showing the occurrence of UbK63 chains in the process of MVB sorting, as well as controversial data obtained in experiments that aimed at defining whether or not modification by these Ub chains plays a role in the sorting process.

### 3.1. Modification of MVB Cargoes by UbK63 Chains: Occurrence, E2s/E3s Involved, and Function of this Modification

In addition to its involvement in the modification of yeast plasma membrane proteins, Rsp5 was shown to be involved in the modification of MVB cargoes [194,195,196]. Given the preference of Rsp5 for the assembly of UbK63 chains [85], the precise mode of ubiquitylation of yeast MVB cargoes was further investigated. The MVB cargo Sna3 was shown to be modified at a single target Lys by UbK63 chains (at least 6–7-residues long), as judged by the modification of the Sna3 ubiquitylation pattern in cells unable to assemble UbK63 chains [57]. Modification of the ubiquitylation pattern in these cells was also reported for other MVB cargoes [15,58] (Table 1). Ubiquitylation involving the addition of UbK63 chains is most likely a very general modification of MVB cargoes coming from either the plasma membrane or the trans-Golgi network: in mutants lacking any of the ESCRT components, these proteins are known to accumulate in an abnormal compartment close to the vacuole known as the class E compartment [197]. This compartment was shown to be immunodecorated by antibodies raised against the K63-Ub linkage, and this labeling was abolished in cells unable to assemble UbK63 chains [58].

What is the function of this modification? In cells expressing UbK63R as a sole source of Ub, several GFP-tagged forms of MVB cargoes were mainly recovered at the vacuolar membrane instead of the vacuolar lumen [14,15,16,58,198], suggesting that modification of cargoes by these Ub chains rather than monoubiquitylation is required for efficient MVB sorting. Consistent with this interpretation, a variant form of the general amino acid transporter Gap1 carrying a single UbK63 di-Ub chain on a unique target Lys was efficiently sorted to MVBs, whereas in UbK63R cells, Gap1 that was monoubiquitylated at two distinct Lys failed to reach this compartment [15]. Moreover, in UbK63R cells, Ub fused in-frame to a GFP-tagged MVB cargo could not re-establish efficient sorting [58]. However, this conclusion was challenged by several observations. For other cargoes, in-frame Ub fusion restored sorting in UbK63R cells [198]. In addition, DUB catalytic domains fused in-frame to several MVB cargoes abolished their ubiquitylation and impaired their MVB sorting. Further fusion of monoUb was then shown to restore MVB sorting [198]. It should be noted, however, that in contrast to similar approaches applied in the study of the internalization step of endocytosis and followed kinetically, all of these experiments relied on the qualitative observation of GFP-vacuolar membrane staining (in case of deficiency) instead of lumenal GFP vacuolar staining (normal phenotype). The setting of more quantitative assays (allowing follow up of the degradation rate) would provide a more accurate understanding of the precise role of modification by permanent monoubiquitylation *versus* modification by UbK63 chains, as well as the potential detection of partial inhibition.

The potential occurrence and role of modification by UbK63 chains in higher eukaryotic cells at MVBs is just emerging. The melanosomal membrane protein MART1/MelanA was shown to interact with two human Rsp5 homologues—Nedd4 and Itch—to undergo ubiquitylation and Ub-dependent MVB sorting [62]. It was further shown to be ubiquitylated by UbK63 chains (immunodetection of ubiquitylated forms by specific antibody) and to accumulate partially at the MVB membrane following the overproduction of UbK63R, which impairs the formation of UbK63 chains [58]. Human endosomes were shown to be the main intracellular compartments that could be immunodecorated with specific antibody directed against UbK63 chains [58,63,199]. A chimeric protein, in which the K63 linkage-specific DUB AMSH was fused to the carboxyl terminus of EGFR, was shown to display reduced K63- and increased mono- and K48-linked ubiquitylation compared with that of wild-type EGFR [64]. EGFR-AMSH was efficiently internalized into early endosomes, but the rates of ligand-induced sorting to late endosomes and degradation of EGFR-AMSH were dramatically decreased compared to that of EGFR [64]. These observations support the hypothesis that UbK63 chains but not multimonoUb constitute a more efficient signal for MVB sorting. As indicated above, similar observations were reported for the plant PIN2 auxin exporter, which was modified by UbK63 chains: a mutant chimeric PIN2^17KR^-Ub was rapidly internalized but displayed a reduced rate of degradation compared to ild-type PIN2 [55].

A spectacular example of the role of UbK63 chains at MVBs was recently provided in the case of worms [72]. In *C. elegans*, fertilization triggers the endocytosis and rapid turnover of maternal surface membrane proteins, including Cav1, the worm homolog of caveolin. The use of anti-UbK63 antibody showed plasma membrane staining after fertilization, followed by a massive transient accumulation of UbK63 chains on endosomes at the one-cell stage [72]. Following fertilization, Cav1 was shown to be modified by UbK63 chains (Table 1), and its polyubiquitylation was strongly impaired in mutants affected in either the E2 Ubc13 or the associated non-catalytic E2 variant Uev1. In these cells, fertilization still led to an accumulation of Cav1 in large endosomes, a process followed by its recycling to the plasma membrane rather than its degradation as in wild type cells. These data suggest that Ubc13-dependent ubiquitylation of Cav1 (and other proteins followed in this study) is dispensable for its internalization but required for lysosomal targeting and associated degradation; thus, it is likely required for MVB targeting [72].

Taken together, these data converge to the hypothesis that modification of MVB cargoes by UbK63 chains resulting from the involvement of various E2s/E3s and often beginning at the plasma membrane plays an important role in efficient MVB sorting.

### 3.2. UBD-Containing Receptors of MVB Cargoes and UbK63 Chains

After the first demonstration that the ESCRT I protein Vps23/Tsg101 is able to bind Ub through its UEV-like domain [186], intensive investigations have shown the presence of UBDs in several proteins of the ESCRT complexes 0–II. Biochemical and biophysical approaches have been used to characterize the interaction of ESCRTs UBD with either monoUb or Ub chains (Box 3) [200]. Strikingly, *in vitro* studies have indicated that most UBDs bind to free Ub with a surprisingly low affinity in the 0.1–1 mM range (Supplementary Table S1) [201]. In contrast, several UBD NZF domains, including that of the ESCRT II protein Vps36, interact with Lys63-diUb with nanomolar affinity (Supplementary Table S1) [202]. Interestingly, proteins belonging to the ESCRT 0 complex contain multiple UBDs (Figure 1 and Box 3). A number of studies, mostly of mammalian proteins, have shown that tandem UBDs permit the recognition of polyUb chains with increased affinity over free Ub recognition (Box 3).

**Box 4. Detection of UbK63 Chains for Lysosomal Targeting**

Structural characterization of the members of the ESCRT machinery in complex with polyUb chains is an ongoing challenge. The ESCRT apparatus contains different complexes [203], which most upstream component is ESCRT-0. The latter one comprises the STAM/Hrs complex [204] in mammalian cells (Vps27/Hse1 complex in yeast) [205]. STAM and Hrs contain six UBDs in total, delineated by two VHS domains (Vps27/Hrs/STAM for STAM and Hrs), a UIM domain (Ub interacting motif for STAM), a double UIM domain (Hrs) and an SH3 domain (Src homology 3 for STAM) [206]. The specific structural organization of these domains ensures that binding of the STAM2 VHS-UIM domains is not only avid but also cooperative with respect to Lys63-Ub_2_ [207]. The VHS-UIM specifically binds Lys63-Ub_2_, with the VHS and UIM domains binding the proximal and distal Lys63-Ub_2_ respectively (Box 3-Figure IB). These results contrast markedly with those obtained for the VHS and UIM domains considered individually [208] and indicate that both these domains are essential for chain specificity. In ESCRT-0, STAM2 uses a combination of multiple UBDs and a cooperative mechanism to increase the binding efficiency of Lys63-Ub_2_ chains. As STAM2 can undergo monoubiquitylation and become inactive, a key question concerns the possible involvement of the same UBDs in this mechanism.

**Figure I:**
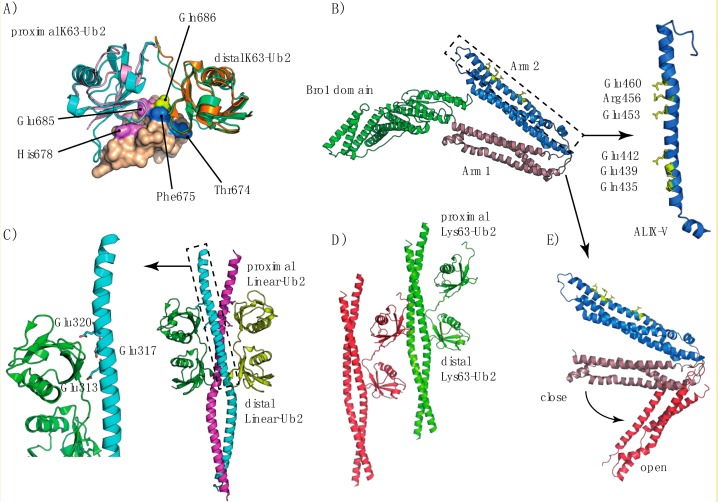
(**A**) Different X-ray structures of the TAB2NZF/Lys63-Ub2 complex, PDB code 3A9J (orange and cyan for the distal and proximal Ub respectively) and PDB code 2WWZ (green and pink for the distal and proximal Ub respectively). The structures are aligned with respect to the TAB2^NZF^ domain (represented by a solvent-accessible surface) of the 3A9J structure. (**B**) Structural organization of the ALIX BRO1-V domains, showing arms1-2 in a close conformation (PDB code 2OEV). The right inset presents an expanded view of the first ALIX-V triad similar to that of NEMO UBAN. (**C**) Structure of the NEMO-UBAN domain in complex with linear-Ub_2_ chains, along with an expanded view of the triad conserved with respect to the ALIX-V domain. (**D**) Structure of the NEMO-UBAN domain in complex with Lys63-Ub_2_ (PDB code 3JSV), in which the Lys63-Ub_2_ proximal domain binds to another NEMO dimer. (**E**) Comparison of the open (PDB code 4JJY) and closed conformations of the ALIX-V domain.

By sharp contrast, the Vps36 subunit of ESCRT-II contains two NZF (Npl4 zinc finger) domains (Figure 1), which enabling ESCRT-II to bind to the Vps28 subunit of ESCRT-I and Ub [209,210]. The NZF domain is also found in other proteins, including TAB2, TAB3 [211], Npl4 [212] and TRABID [213]. Vps36^NZF2^ binds Lys63-Ub_2_ chains more tightly than linear Ub_2_ or Ub (Supplementary Table S1), whereas TAB2 and TAB3 specifically bind Lys63-Ub_2_ [160,211]. In the absence of information about the structure of the Vps36^NZF2^/Lys63-Ub_2_ complex, the origins of this difference remain unclear. The structure of the TAB2^NZF^/Lys63-Ub_2_ complex provides some useful information. Indeed, important residues involved in the interaction are conserved within the Vps36^NZF2^ sequence [155]. At first sight, Lys63-Ub_2_ adopts a conformation in which the two Ub moieties surround the TAB2^NZF^ domain (Figure IA), binding to each other via their hydrophobic patches. Proximal Ub recognition is mediated by the conserved residues His678 and Glu685 (His191 and Glu198 in Vps36^NZF2^), whereas Thr674 and Phe675 (Thr187 and Phe188 for Vps36^NZF2^) interact with the distal Ub. By contrast to Vps36^NZF2^, the chain-selective binding of TAB2^NZF^ seems to be related to the hydrophilic Gln686 residue (Ile199 in Vps36^NZF2^) located in the Φ position in the TF/Φ motif for Ub binding.

In yeast, Ub recycling is dependent on Bro1, which recruits the deubiquitylating enzyme Doa4 to ESCRT-III [184]. Alternatively, Bro1 may function as a Ub receptor in addition to ESCRT-0 for protein sorting into multivesicular bodies (MVBs) [214]. In mammalian cells, Alix plays a similar role to Bro1, binding to Ub and polyUb chains via its V-domain [73,215]. Our vision of protein sorting into MVBs may therefore be enlarged by the presence of adaptor proteins—ALIX and HD-TP—which bind directly to Ub or to the cargo protein. Biosensor analysis has shown that ALIX^V^ preferentially binds to polyUb chains containing at least four Lys63-linked Ub molecules [73]. Interestingly, ALIX^V^ contains two Glu-rich triads (Figure IB), which have been predicted to contribute to linkage-specific polyUb binding [73] and participate in the interaction with Ub [215]. Further studies of the structure of ALIX have shown that the ALIX^V^ domain has conserved sequence residues also common to the NEMO^UBAN^ domain (Ub-binding in ABIN and NEMO), encompassing the CC2 (coiled coil) and LZ (leucine zipper) domains, and consisting of a parallel dimeric coiled coil that preferentially binds linear polyUb chains [216]. These chains have an open conformation and inter-Ub distance similar to that of Lys63-Ub_2_ chains [160] (Figure IC,D). In addition, Laplantine and coworkers have reported that the addition of the NEMO-ZF (zinc finger) domain results in an affinity for UbK63 about 100 times greater than that of K48Ub chains [217].

Bro1 belongs to a larger family of related proteins, including the mammalian Alix and HD-PTP, with a common architecture. These proteins bind ESCRT-I, and have both an N-terminal Bro1 homology domain that binds the ESCRT-III subunit Snf7/CHMP4 and a middle V domain that, in the case of Alix, binds YPxL peptide motifs [218,219,220]. The characteristic V-domain is organized into two trihelical bundles forming a V-shape, with a short and a long arm (Figure IB). Most studies of the structure of Alix and Bro1 have reported a closed conformation of these arms, restricting access to potent ubiquitylated proteins. However, small-angle X-ray scattering (SAXS) data have revealed a possible open conformation of the Alix V-domain (Figure IE). Alix can undergo automonoubiquitylation [220] and this process may be achieved by the binding of the Alix-V domain to the Ub moiety, generating a closed, inactive conformation.

**Figure II:**
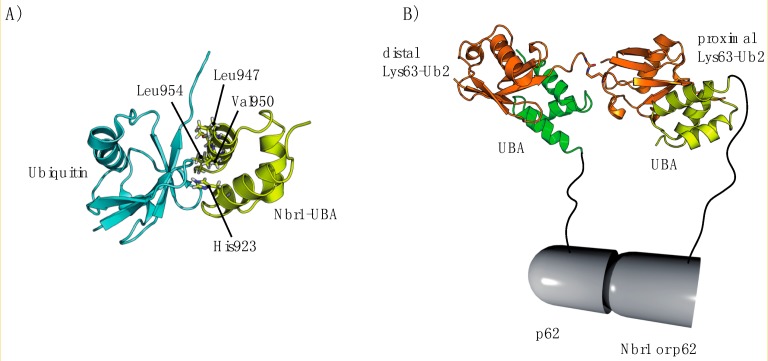
(**A**) Structure of the Nbr1-UBA domain in complex with Ub (PDB code 2MJ5), showing the UBA-interacting residues. (**B**) Putative model explaining the affinity of p62 or Nbr1 for Lys63-Ub2 chains. Upon binding via the PB1 domain, dimerization may favor the binding of the p62- or Nbr1-UBA domains to Lys63-Ub2. The structure of the Nbr1^UBA^/Ub complex has been superimposed on the structure of Lys-Ub2 (PDB code 2JF5).

In the context of autophagy, both p62 and Nbr1 are cargo adaptors with a UBA domain at the extreme *C*-terminus (Figure 1) for the recognition of polyubiquitylated proteins [221]. Indeed, p62 has a higher affinity for UbK63 chains than for UbK48 chains if theSer403 residue is phosphorylated [222]. A key structural feature of p62 is its ability to homodimerize via its PB1 domain [223], potentially leading to linkage-specific avidity for UbK63 chains, through the provision of several Ub-associated (UBA) domains for Ub interaction. Another layer of complexity has been attributed to this system with the possible dimerization of the p62-UBA domain and the repression of Ub binding, whilst association with Lys63-linked chains is favored [224]. The p62-UBA domain exists in solution as a monomer/dimer equilibrium (Supplementary Table S1) and its structural organization has been elucidated both by NMR and X-ray methods [225,226]. The structure of the p62/Lys63-Ub_2_ complex has not yet been determined, but a possible mechanism can be inferred from the recent structural data published for the Nbr1 protein. Indeed, the Nbr1-UBA domain shows affinity for both Lys63- and Lys48-linked polyUb chains and might undergo a conformational change to expose its UBA domain [227]. Nbr1^UBA^ folds similarly to p62^UBA^ and exists in a monomer-dimer equilibrium, with a weaker affinity than p62 [228] and a different dimerization surface. The resulting Nbr1^UBA^/Ub structure (Figure IIA) resembles the DSK2^UBA^/Ub complex, with conserved interfacial residues [228], except that Nrb1^UBA^ recognizes Ub chains in a linkage non-specific manner (Supplementary Table S2). Merging the information available for p62^UBA^ and Nbr1^UBA^ domains, a plausible mechanism has been proposed to account for the preference for polyUb, in which the full-length Nbr1 binds to p62 via its *N*-terminal PB1 domain. In this structural organization, a Lys63-Ub_2_ chain could accommodate two UBA domains (Figure IIB), as previously described for the UBA domain of ubiquilin-1 [229].

If the functions of the core ESCRT 0, I, II and III complexes are conserved from yeast to man, the ESCRT-associated proteins Bro1 in yeast and its mammalian homolog ALIX [apoptosis-linked gene 2- interacting protein X] (Box 4) share a similar structural organization but were initially shown to have distinct properties. Both proteins interact with the ESCRT III protein Chm4/Snf7 via their Bro1 domain [192,230], but their Pro-rich domains (PRDs) have distinct partners: the ESCRT I protein Tsg101 for ALIX [192], and the DUB Doa4 for Bro1 [230]. ALIX was initially shown to be dispensable for MVB sorting of EGFR, whereas, in addition to its function in Doa4 localization, Bro1 was shown to be required for the maximal efficiency of ILV budding [231]. The V domains of both Bro1 and ALIX were shown to act as a new UBD [73,214,215]. In the case of ALIX, preferential binding to UbK63 chains was observed (Box 4) [73,215]. A genetic interaction between deletion of *BRO1* and hypomorphic mutations in ESCRT 0 led to the discovery that, in addition to ESCRT 0 proteins, Bro1 may function as a Ub receptor that functions in early steps of protein sorting into MVBs [214]. It is tempting to think that both Bro1 and ALIX contribute to a more efficient sorting of MVB cargoes that are modified by UbK63 chains.

Interestingly, plants are devoid of ESCRT 0 complexes. They do, however, possess TOL (TOM-like) proteins, which are involved in vacuolar sorting [232]. Interestingly, TOL proteins also have a modular structure with tandem UBDs: a *N*-terminal VHS followed by a GAT domain. TOL proteins appear to have functions similar to ESCRT 0 [232], and it can be hypothesized that, similarly to the situation in STAM (Figure 1 and Box 3), the tandem organization of their UBDs is responsible for a high-affinity binding to UbK63 modifications of proteins, such as PIN2.

As described above, many UBD-containing proteins undergo ubiquitylation, and some mammalian ESCRT were among the first examples of this property [169,233]. The ESCRT 0 proteins Hrs and STAM were shown to be hyper-ubiquitylated in cells deficient for the UbK63-specific DUB AMSH [234], suggesting that they might be modified by UbK63 chains. A systematic analysis of the potential ubiquitylation status of all yeast UBD-containing ESCRTs was undertaken by epitope tagging of these proteins at the chromosomal locus and expression of His-tagged Ub. All of these proteins were shown to be ubiquitylated in an Rsp5-dependent manner, and some of them displayed Ubp2-dependent ubiquitylation, suggesting that a fraction of these proteins is modified by UbK63 chains [58].

Whether ESCRT ubiquitylation regulates MVB sorting remains an open question. In yeast, individual fusion of a DUB catalytic domain to ESCRT-0 or I proteins abolishes their ubiquitylation but has no effect on targeting to the vacuole of MVB cargoes permanently fused to Ub [198]. Whereas these data argue against a regulatory function of ESCRT 0 or ESCRT I ubiquitylation in the sorting process, it is possible that ubiquitylation of ESCRTs other than the one fused to the DUB domain is maintained and could play a role in the sorting process.

Strikingly, the average size of ILVs is reduced in *rsp5* compared to wild-type cells [235], and yeast cells deficient in UbK63 chain assembly display a defective MVB ultrastructure, *i.e.*, remnant MVBs with fewer ILVs [58]. An altered MVB morphology was also observed in mammals after down-regulation of the E3 Triad, which interacts with both K48- and K63-linkage-specific E2s [66]. These effects on MVB biogenesis could result from defective ubiquitylation of either cargoes, the ESCRT machinery, or both, arguing that UbK63 chains are important for MVB sorting. In agreement with a critical role of cargo ubiquitylation, it was reported that expression in yeast of a chimeric Hse1-DUB completely abolishes ILV formation, a process that is partially restored by expression of a permanently fused ubiquitylated cargo [236]. However, studies of endosomal DUBs (see below) support a role for UbK63 chains in the function of ESCRT. Hence, the validation of a potential function of ESCRT modification, notably by UbK63 chains, awaits further investigations.

### 3.3. DUBs Specific against UbK63 Chains and Their Role at MVBs

Several DUBs that disassemble UbK63 chains display a functional connection with MVB sorting: Ubp2 in *S. cerevisiae*, AMSH in mammals (Figure 1 and Box 5) and plants, an AMSH homolog in *Schizosaccharomyces pombe*. Strikingly, mutations in AMSH were shown to lead to a congenital human disorder, microcephaly-capillary malformation [237].

There is currently little information on the function of Ubp2. It was shown to interact with Rsp5 via a common partner, Rup1 [61,238], and to interact indirectly with the ESCRT 0 protein Hse1. *ubp2Δ* cells were shown to display normal plasma membrane ubiquitylation of the uracil permease Fur4, delayed vacuolar targeting and degradation of this protein [239], and a slight MVB sorting defect of Cps1 (carboxypeptidase S) [238]. It is unlikely that the latter defect results from increased Cps1 ubiquitylation, which would have the opposite effect. The effect appears to be more consistent with the observed increase in the ubiquitylation status of ESCRT proteins in *ubp2Δ* cells, notably that of Hse1 [58], and thus suggests a regulatory role of Ubp2 on this ESCRT protein. Alternatively, the effect could correspond to a regulation of the activity of Rsp5 itself, which was shown to display self-ubiquitylation controlled by Ubp2 [239].

**Box 5. Disassembling UbK63 Chains**

**Figure I:**
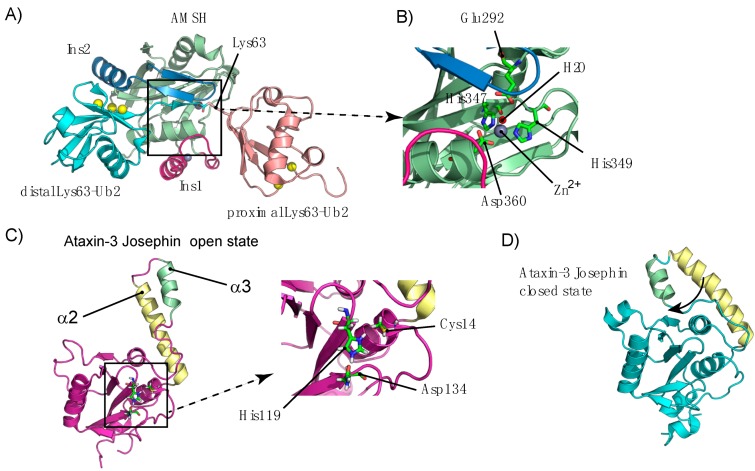
(**A**) Structure of the AMSH-LP/K63-Ub_2_ complex (PDB code 2ZNV) showing the JAMM core Ins1 and Ins2. The distal and proximal domains of K63-Ub_2_ are shown in cyan and pink, respectively, whereas the Ub hydrophobic patch defined by Val70, Ile44 and Leu8 is represented as yellow spheres. (**B**) The inset represents an expanded view of the catalytic region of AMSH and the coordination of the Zn^2+^ ion. (**C**) Structure of the open conformation of the Ataxin-3 Josephin domain (PDB code 2JRI), together with an expanded view of the catalytic triad. (**D**) Structure of the closed conformation of the Ataxin-3 Josephin domain (PDB code 2AGA).

The removal of Ub or polyUb tags is mediated by deubiquitylating enzymes (DUBs), including AMSH and UBPY, which are involved in lysosomal degradation. AMSH (associated molecule with a Src homology 3 domain of signal transducing adaptor molecule -STAM) is a member of the JAMM (JAB1/MPN/MOV34) family of deubiquitylating enzymes [233,240,241], whereas UBPY (Ub-specific protease Y), also known as USP8, belongs to the Ub-specific protease (UBP) family of cysteine proteases [242].

Both AMSH and UBPY, have been shown to associate directly with the early ESCRT-0 complex through the SH3 binding motif (SBM) of the STAM protein [234,243,244], as well as with chromatin-modifying protein (CHMP) components of the late ESCRT-III machinery [245,246,247,248]. Several lines of experimental evidence have revealed roles for AMSH and UBPY at different levels, but their precise involvement in terms of localization, mechanism of action and temporal efficiency remains elusive, mostly due to the absence of clear structural details. In addition to belonging to different families, AMSH and UBPY have different specificities for polyUb chains, as the JAMM metalloprotease AMSH preferentially cleaves UbK63 chains [233], whereas UBPY presents no such selectivity for chain cleavage. Probably the most cogent illustration of a DUB/Lys63-linked polyUb complex to date has been provided by the structure of the AMSH-LP catalytic core in complex with Lys63-Ub_2_ (Figure IA,B). AMSH and AMSH-LP have JAMM domain sequences that are 54% identical and 75% similar [249]. They are folded similarly, but differ in stability. The distal Ub binds to a specific insertion region, corresponding to residues 314–339 (302–327 in AMSH), allowing the proximal Ub residues Gln62, Lys63 and Glu64 to align correctly for linkage-specific deubiquitylation (Figure IA). The interaction with the proximal Ub also facilitates the correct orientation of the proximal Ub and the accurate positioning of the isopeptide-linked Lys63 for efficient deubiquitylation. ITC experiments have clearly shown that AMSH does not distinguish between the polyUb substrate and its Ub product (Supplementary Table S1) but that it interacts directly with the distal hydrophobic patch. Further support for this structural organization has been provided by the structure of the Sst2/Ub complex, determined for the Sst2 ortholog of AMSH crystallized with a Ub product [250]. AMSH-LP has no functional SBM or MIT (microtubule interacting and transport) domain, and therefore cannot bind to other components of the ESCRT machinery, but a host of experimental studies have shown that the STAM protein connected to AMSH in a more subtle manner and serves as an activator of this enzymatic process [245,249], with the UIM domain of STAM serving as an activator in interactions with the proximal Ub of Lys63-Ub_2_ and AMSH interacting with the distal Ub. The complete mechanism underlying the cleavage of UbK63 chains and involving AMSH, STAM and Lys63-Ub_2_ has yet to be elucidated.

Ataxin-3 belongs to the Josephin DUB family (Figure 1). It recognizes both UbK63 and UbK48 polyubiquitin chains through the cooperative activity of its two UIM domains [251,252]. It consists of an *N*-terminal globular Josephin domain and a flexible *C*-terminal tail containing up to three UIM domains and a polyQ tract [253]. The catalytic site is built around a triad of residues Cys14, critical for Ub catalytic activity, His119 and Asp134. Ataxin has been shown to act as a “barber”, maintaining the correct length of the polyUb chains on target proteins and thereby determining their fates [83]. Furthermore, its catalytic activity is directly regulated by ubiquitylation [254]. UbK48 and UbK63 and mixed-linkage polyUb chains bind Ataxin-3, but Lys63- and Lys48/Lys63-mixed linkages are preferentially cleaved *in vitro* [255]. The UIM domains mediate the binding of Ub chains [166,255], but two additional binding surfaces have been identified on the Josephin domain (Figure IIA) [251]. These different sites have been shown to be essential for polyUb chain cleavage by Ataxin-3. The specific role of the Josephin domain in cleavage has been investigated by a combination of NMR and molecular docking [256]. A putative model of the possible interaction of Lys48- or Lys63-Ub2 chains with the Josephin domain of Ataxin-3 developed by Nicastro and coworkers indicated that Lys48-Ub_2_ could occupy both sites simultaneously, whereas Lys63-Ub_2_ could not [256]. The Josephin fold has a specific feature in the form of a helical hairpin, containing helices α2 and α3 (Figure IC). This hairpin structure is close to the active site and is therefore likely to be directly involved in the cysteine protease activity of the domain and to be responsible for accommodating polyUb chains and other molecular partners (Figure IC,D). The UIM domains are also an important feature of Ataxin-3 for polyUb chain cleavage. Indeed, the structural organization of the UIM12 domains is likely to be flexible in the free state, whereas these domains adopt a compact structure when bound to Ub (Figure IIB). Moreover, cooperative effects may be observed during the binding of Ataxin-3^UIM12^ to Lys48- or Lys63-linked chains, potentially favoring cleavage by the Josephin domain. The UIMs may help to recruit polyubiquitylated substrates, to position the polyUb chain relative to the catalytic site, or may enable the enzyme to trim polyUb chains in a distal-to-proximal direction. This strongly suggests that Ataxin-3 functions as a poly-Ub-specific “molecular ruler”, selecting poly-Ub chains of the correct length.

**Figure II:**
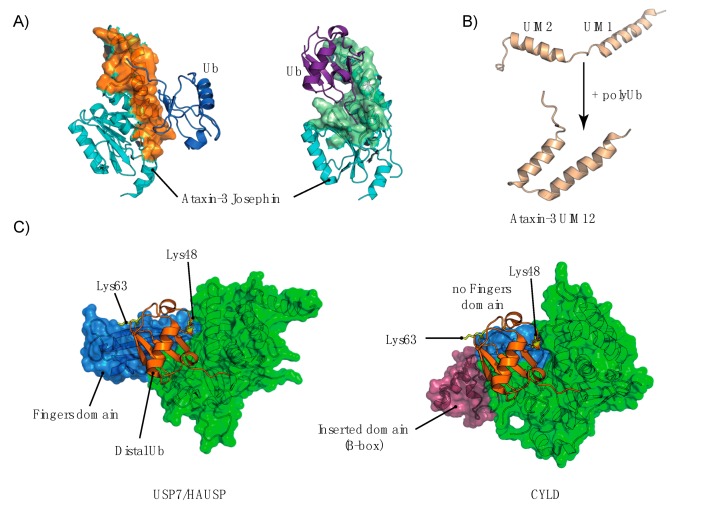
Structures of the Ataxin-3 Josephin/Ub (**A**) binary complexes, showing the two binding sites available on the Josephin domain. (**B**) Upon binding to polyUb chains, the Ataxin-3^UIM12^ domains adopt a compact structure (PDB code 2KLZ). (**C**) Structure of USP7/HAUSP (PDB code 1NBF) and CYLD (PDB code 2VHF) in complex with Ub. The positioning of Ub on CYLD arises from the structural alignment of USP7/HAUSP-Ub on CYLD. The absence of Fingers domain and an extended loop near the catalytic domain renders CYLD specific toward UbK63 chains and prevent UbK48 from binding.

While UBPY (USP8) and CYLD belong to the Ub-specific proteases family (USPs), they differ by their specificity and accessibility for polyUb chains. Ub binding surface loops occlude the active site in the case of UBPY [257] whereas the USP domain of CYLD is accessible for catalysis [179]. The specificity of CYLD for UbK63 chains has been recently ascribed to its specific structural features [179]. More precisely, CYLD harbors an extended loop near the active site (Figure IIC). Other USP domains that have a significant shorter loop do not show any specificity for UbK48 or UbK63 chains. Additionally, CYLD exhibits a shorter Fingers subdomain, which renders Lys63 accessible and allows chains elongation toward the distal end, while Lys48 would be occluded.

Mammalian AMSH (Figure 1) was initially identified as a partner of STAM [240]. Subsequently, its interaction with several ESCRT III proteins was reported [258], demonstrating that this DUB is deeply embedded in the MVB sorting machinery interaction network. In agreement with these interactions, AMSH was shown to be located at endosomes, in addition to its localization to the nucleus and cytoplasm [233]. AMSH belongs to the JAMM (JAB1/MPN/MOV34 metalloenzymes) domain metalloprotease family of DUBs and carries a microtubule-interacting and transport domain (MIT) that is responsible for its interaction with ESCRT III proteins. AMSH was shown to display deubiquitylation activity *in vitro*, specifically against UbK63 chains [233], an activity that was stimulated by STAM [245]. In *S. pombe*, a connection between MVB sorting and AMSH was established thanks to a genetic screen: a sterility defect due to the loss of Ste12 phosphatidylinositol 3-phosphate 5-kinase (Fab1 in *S. cerevisiae*), which is critical for MVB formation, was suppressed by the mutation of several genes encoding either ESCRT components or AMSH [259]. In mammals, several studies report an accumulation of ubiquitylated proteins at endosomes after down-regulation of AMSH [233,260]. The experimental data used to identify the *in vivo* the functions of mammalian AMSH at endosomes resulted in complex, sometimes contradictory, information, possibly as a result of the different targets of AMSH in different cell lines or systems. AMSH was shown to deubiquitylate *in vitro* EGFR immunoprecipitated from EGF-stimulated cells [233], and siRNA knockdown of AMSH resulted in accelerated EGFR degradation in HeLa cells, which suggests that AMSH-dependent deubiquitylation of EGFR counteracts Cbl-dependent ubiquitylation of the receptor [233]. In contrast, expression of an inactive dominant-negative (DN) form of AMSH in the same cells inhibited EGF-induced EGFR degradation [258]. This was explained by an increased binding of DN-AMSH to ESCRT III components, preventing MIT-dependent binding of the Vps4 ATPase required for disassembly of the ESCRT III complex and completion of the sorting process [258].

In addition to the potential role of AMSH in the deubiquitylation of MVB cargoes or in Ub-independent interference with the disassembly of the ESCRT III complex, the role of AMSH at MVBs appears to be an effect on the sorting machinery, as revealed in studies of its involvement in the intracellular fate of the G protein-coupled receptor CXCR4, which undergoes both constitutive and ligand-induced turnover [234]. AMSH depletion or overexpression of dominant negative (DN) AMSH results in the accumulation of ubiquitylated forms of the ESCRT 0 proteins STAM and Hrs and increased steady-state levels of CXCR4 in different human cell lines. This effect was abolished following the expression of DN AMSH unable to interact with STAM [234]. These observations suggest that AMSH-dependent STAM and Hrs ubiquitylated status (presumably with UbK63 chains) regulate the function of ESCRT 0 in MVB sorting of CXCR4 and potentially of other MVB cargoes [234].

Plant cells have three AMSH homologs: AMSH1, AMSH2 and AMSH3. AMSH3 was shown to deubiquitylate UbK63 and UbK48 chains *in vitro*. *AMSH3* null mutants display defects in vacuole biogenesis and intracellular trafficking. AMSH3 was shown to bind several ESCRT III components of *Arabidopsis* *thaliana* and to be required for their proper localization [193]. *AMSH3* cells accumulate ubiquitylated membrane-bound proteins and display reduced degradation of an endocytic substrate, an observation suggesting its involvement in the MVB sorting machinery rather than in deubiquitylation of MVB cargoes [261]. These interesting data must be complemented by the identification of AMSH3 plant substrates.

Taken together, these data suggest a complex involvement of AMSH homologs, which influence, sometimes in an opposite way, MVB sorting *via* deubiquitylation of MVB cargoes and/or components of the ESCRT machinery.

### 3.4. UbK63 Chains and Viral Budding

Enveloped viruses escape infected cells by budding away from the cytoplasm in a process with a similar topology to ILV formation at MVBs. Thus, it was not surprising to discover that enveloped viruses can usurp part of the MVB sorting machinery (several ESCRT I and III components, Alix) and some E3 ligases of the Nedd4 family to allow their budding at the plasma membrane (or sometimes in intracellular compartments). Retroviruses recruit the ESCRT machinery through late assembly (L) domains in the viral polyprotein Gag. HIV-1 Gag harbors two types of L domains, a PTAP motif that binds to the ESCRT I protein Tsg101, and a LYPx_n_L-type that binds the V domain of ALIX (recently reviewed in [262]). Viral budding of other retroviruses involve PPxY L-type domains in Gag proteins, which interact with WW domains of Nedd4 family members. The observation that overexpression of UbK63R (and not UbK48R) leads to a modest inhibition of the formation of virus-like particles was the first hint of a potential role of UbK63 chains in viral budding [263]. An HIV-1 construct lacking the PTAP motif of Gag is poorly released. The search for factors that, when overexpressed, rescue the release of this mutant HIV-1 constituted a powerful tool to identify important factors in viral budding [74,264]. Overexpression of the active form of the HECT E3 ligase NEDD4-2 stimulated the release of HIV-1 constructs lacking the Gag PTAP motif. Rescue was also observed following the overexpression of chimeric HECT domain E3 ligases and even isolated catalytic domains, provided that they were able to assemble UbK63 chains [74]. Gag ubiquitylation was not sufficient to promote viral release rescue [74], and thus, the ubiquitylation of other proteins was required, possibly that of Tsg101, which was shown to be ubiquitylated following the overexpression of Nedd4-2 [264].

Further evidence for the correlation between viral budding and UbK63 chains was obtained from the study of Alix Ub binding. As stated above, the ALIX V domain was shown to preferentially bind to UbK63 chains that were at least four-Ub long [73] (Box 4). Overexpression of wild type Alix, but not of Alix containing a mutation in its UBD, stimulated the release of the HIV-1 PTAP mutant [73,215], further supporting a role for UbK63 chains in the budding process. The ubiquitylated proteins bound by ALIX during viral budding remain to be identified.

## 4. Modification of Proteins by UbK63 Chains at Other Trafficking Steps in the Endocytic Pathway, or during Recycling Events

### 4.1. The Role of Ras Ubiquitylation in Its Trafficking

Ras proteins are small GTPases that regulate cell growth, proliferation and differentiation. The different Ras isoforms -H-Ras, N-Ras and K-Ras- generate distinct signal outputs. H-Ras and N-Ras are palmitoylated at cysteine residues. The latter modification is required for the entry of these proteins into the exocytic pathway and their trafficking from the Golgi to the internal leaflet of the plasma membrane. H-Ras and N-Ras undergo internalization from the plasma membrane to endosomes, followed by recycling to the Golgi apparatus. They are present at steady state in the Golgi and endosomes, and they mediate slower, sustained signaling from their intracellular locations compared with plasma membrane K-Ras (reviewed in [265]).

The trafficking of these two Ras isoforms is influenced not only by their palmitoylation but also by their ubiquitylation. Overexpression of various Ub forms (WT, UbK0, UbK48R, UbK63 only) provided evidence that H-Ras and N-Ras undergo mono- and mainly di-ubiquitylation, with extension by UbK63. This ubiquitylation is constitutive, depends on Ras palmitoylation, and alters Ras localization: a mutant H-Ras lacking all lysines (HRas8RK) is not ubiquitylated and is predominantly located at the plasma membrane and enriched in the Golgi apparatus, whereas its endosomal pool disappears. Conversely, a chimeric Ub-H-Ras displays enrichment at early endosomes and a reduced presence in the Golgi pool [266]. Ubiquitylation of H-Ras affects its signaling properties: H-Ras8RK is more efficient and Ub-H-Ras is less efficient for Erk activation [266]. These results demonstrated that di-ubiquitylation of H-Ras with UbK63 chains is important for its trafficking from the Golgi to endosomes or for retention on early endosomes.

Biochemical and genetic studies simultaneously led to the identification of the E3 involved in H-Ras ubiquitylation [67,267]. Xu *et al.* focused on Rabex-5, the guanine nucleotide exchange factor (GEF) for Rab5, because it was previously shown to interact with H-Ras [268]. In addition to its GEF activity mediated by a Vps9 domain, Rabex-5 possesses E3 Ub ligase activity that is mediated by an amino-terminal zinc finger (ZnF) domain belonging to the A20 ZnF family. Indeed, overexpression of Rabex-5 was shown to stimulate mono- and di-ubiquitylation of H-Ras, and Rabex-5 silencing suppressed H-Ras ubiquitylation [67]. Overexpression of Rabex-5 triggered an increase in the endosomal pool of H-Ras and inhibition of H-Ras-mediated signaling [67]. In complete agreement with these observations, *Drosophila* Rabex-5 was identified as a negative regulator of Ras and was shown to mediate drosophila Ras ubiquitylation with the same characteristic di-ubiquitylated form [267].

Interestingly, current knowledge regarding Ras ubiquitylation by Rabex, a diubiquitylation process involving UbK63, raises numerous questions. What are the structural properties that lead to preferential ubiquitylation by Rabex with UbK63? Why is the modification limited to mainly di-ubiquitylation? Which E2 acts *in vivo* with Rabex? Because H-Ras undergoes apparent constitutive ubiquitylation, internalization, and endosome-to-Golgi recycling, does ubiquitylation then drive Golgi-to-endosome trafficking, or is it simply required for endosomal retention? Finally, what is the fate of ubiquitylated Ras: is it deubiquitylated for recycling to the plasma membrane, or is it targeted to MVBs and subsequently degraded [269]?

### 4.2. Role of UbK63 in Sorting from Rab10 Endosomes

AMPA-type glutamate receptor (AMPAR) trafficking plays a key role in synapse strength and plasticity. A genetic screen for mutants with impaired trafficking of the AMPAR subunit GLR1 in *C. elegans* led to the identification of a crucial role for Uev1 [270]. GLR1 is endocytosed by both clathrin-dependent and clathrin-independent mechanisms, and Rab10 was shown to be involved in the recycling back to the synaptic membrane of receptors endocytosed by the latter mechanism. Both *uev1* and *ubc13* mutants display defective Glr1 localization: the receptor accumulates in elongated accretions in neuron cell bodies where it colocalizes with Rab10. The prevention of direct ubiquitylation of Glr1 (important for its endocytosis) does not suppress the *uev1* phenotype, suggesting that modification by UbK63 chains of other proteins is required for the exit of Glr1 from the Rab10 compartment.

### 4.3. A New Paradigm in UbK63-Mediated Ubiquitylation: WASH Activation by Ubiquitylation

The rapid polymerization of actin to create patches of actin filaments was first shown to be required for the internalization step of endocytosis and later for other trafficking steps, including endosome-to-Golgi recycling. Actin polymerization at multiple cellular sites requires the Arp2/3 complex together with a nuclear-promoting factor (NPF). The prototypical actin NPF is the Wiskott–Aldrich syndrome protein (WASp), which functions in actin polymerization at the plasma membrane. WASp homologs include WASH1, which is located at endosomes. WASH exists in a macromolecular complex named WASH Regulatory Complex (SHRC) that consists of five core factors including FAM21. SHRC is recruited to early endosomes by interactions between FAM21 and Vps35, a subunit of the retromer complex. Both SHRC and the retromer are required for several recycling routes, including endosome-to-Golgi retrieval of the cation-independent mannose 6-phosphate receptor (CIMPR) [271].

The WASp family of NPFs is defined by the presence of a VCA domain at the *C*-terminal end of the protein that is critical for the process of Arp2/3-mediated actin polymerization. Conformational changes in the actin NPF regulate access of the VCA domain to Arp2/3 and thereby control the activity of the respective actin NPF. Several signaling molecules have been shown to promote the activation/exposure of WASP VCA motifs to allow timed and localized F-actin nucleation [272], but the mechanisms regulating WASH activation have remained elusive.

Deciphering the mechanisms underlying WASH activation and the role played by Ub in this process arose from studies that were initially unrelated to trafficking. The story began with an attempt to identify the function(s) of a family of proteins that are similar to melanoma antigen (MAGE). A biochemical search for binding partners revealed that MAGE proteins bind to and activate in a specific manner TRIM E3s RING finger enzymes [273]. Hao and coworkers investigated the potential function of the MAGE-L2/TRIM27 complex. They observed that both proteins localize to endosomal structures and showed that MAGE-L2 binds to the retromer partner Vps35. Both MAGE-L2 and TRIM27 were required for CIMPR endosome-to-Golgi retrieval. Downregulation of the intracellular pool of UbK63 chains specifically inhibited this trafficking step of CIMPR. siRNA of MAGE-L2, TRIM27 or down-regulation of UbK63 chains impaired endosomal F-actin assembly. The target of MAGE-L2/TRIM27-dependent ubiquitylation was shown to be WASH, which undergoes modification by UbK63 chains at K220. Reconstitution of WASH ubiquitylation and actin assembly from purified components finally suggested that WASH ubiquitylation at K220 facilitates WASH activation by allowing WASH VCA exposure [69]. UbK63-dependent ubiquitylation thus acts as a molecular switch that allows the conformational changes required for WASH activation. The event that triggers WASH ubiquitylation and activation, and whether deubiquitylation events also regulate the process remain to be elucidated.

## 5. Role of UbK63 Chains in Trafficking Events Associated with NFκB Signaling Pathways

One important function of UbK63 chains, which was observed years ago, is their involvement in activation of kinases in various signaling pathways, most notably the NFκB pathways, which play a critical role in a large number of normal and pathological processes including inflammation and the immune response*.* This field has been the subject of detailed investigations that are beyond the scope of this review (see for recent review [274]). Here, we will only highlight recent reports describing some trafficking events that are dependent on the formation of UbK63 chains associated with the correct functioning of NFκB signaling.

In resting cells, the nuclear factor family of transcription factors NFκB is sequestered in an inactive form in the cytoplasm *via* interactions with inhibitors (IκB). In response to various signals, IkB is phosphorylated by a cytosolic kinase complex, IKK, which consists of two kinase subunits, IKKα and IKKβ, and a regulatory subunit, NFκB essential modulator (NEMO). Phosphorylated IkB is then ubiquitylated and degraded by the proteasome, thus allowing NFκB to translocate to the nucleus and regulate the expression of numerous genes. The most well studied pathways of inflammatory cytokine-induced NFκB activation are those stimulated by interleukin 1 (IL-1) and tumor necrosis factor alpha (TNF-α). IL-1 and TNF-α bind to their respective receptors, IL-1R and TNFR, and trigger activation of diverse signaling cascades that converge at TGF-α activated kinase 1 (TAK1), which phosphorylates and activates IKK. Stimulation of cells by IL-1 leads to the activation of the E3 TRAF6, which together with Ubc13/Uev1 catalyzes the assembly of UbK63 chains on itself and on the IL-1 receptor (IL-1R)-associated kinase 1 (IRAK1), a kinase that is recruited to ILR1. The resulting UbK63 chains recruit the TAK1–TAB1–TAB2/3 complex and the IKK complex via UbK63 selective binding of TAB2/3 or NEMO, respectively. Indeed, NEMO bipartite UBD (UBAN plus ZnF) binds specifically to UbK63 chains [217]. TAK1 then phosphorylates IKKβ, thus activating the IKK complex. The essential role of UbK63 chains in the IL-1R pathway was clearly established by showing that replacement of endogenous Ub with UbK63R in human cells impaired IL1-induced signaling [102]. The same study also indicated that, in contrast, UbK63 chains appeared to be dispensable for activation of the pathway by TNF, which involves other factors. Growing evidence has revealed that in addition to UbK63 chains, other types of Ub chains play a role in NFκB activation in response to cytokines, notably linear chains [275]. In addition, recent data have shed light on a less expected involvement of UbK63 chains in the NFκB pathway: regulation of the subcellular localization of specific components.

In resting cells, the NEMO-IKK complex resides in the cytoplasm. Immunofluorescence experiments performed using cells treated with saponin to extract cytoplasmic NEMO revealed that IL-1 induces the rapid and transient (up to 15 min) recruitment of NEMO into punctate structures anchored at the cell periphery at sites distinct from IL-1R. These NEMO foci also contain Ub and IRAK1. The formation of these structures was abolished in cells that were unable to assemble either UbK63 chains or linear Ub chains, or in situations in which NFκB signaling was impaired [276]. It was proposed that this cytokine-induced local accumulation of NEMO in association with IKK kinases is maintained by Ub chains acting as a scaffold prior to being dissociated by the involvement of the various DUBs, which disassemble UbK63 chains (CYLD and A20) or linear chains (OTULIN) [276]. Among the multiple questions raised by these observations, it remains to be determined how the NEMO foci are linked to the plasma membrane.

The mechanism by which NFκB activation is transmitted to the cell interior remains ill-defined. A biochemical approach permitted the identification of a critical component involved in this process. Total membranes were prepared before or after stimulation of the pathway *via* different receptors (TNFR, CD40, *etc.*), fractionated into different organelles, and analyzed by western blot for the presence of various components of the pathway. After stimulation, the endoplasmic reticulum (ER) was found to be enriched in ubiquitylated forms of various components of the pathway. Immunoprecipitation of these components followed by mass spectrometry analysis revealed that the ubiquitylated proteins were retained by metadherin (MTDH), an ER-anchored protein with increased expression in several carcinomas. Down-regulation of MTDH impaired NFκB signaling. GST-MTDH was found to directly bind UbK63 chains. Thus, it was proposed that MTDH operates as a scaffold that retains, at the cytoplasmic leaflet of the ER, ubiquitylated participants in the pathway that are involved in NFκB signaling, notably the receptor-interacting Ser/Thr protein kinase 1 RIP1, modified by UbK63 after NFκB stimulation. This step acts downstream of the stimulation of multiple immunoreceptors and specifically involves interactions with the ER membrane of UbK63-modified proteins, which are required for the normal activation of NFκB [277].

## 6. Roles of K63-Linked Ubiquitylation in Selective Autophagy

**Figure 3:**
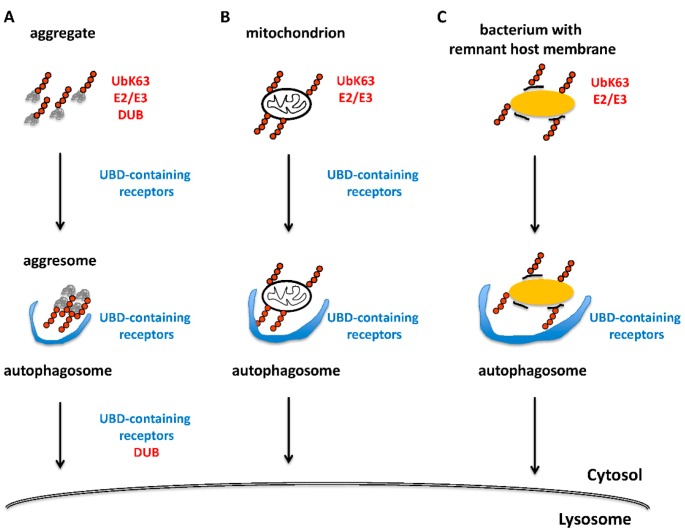
Involvement of UbK63 chains in selective autophagy.

Macroautophagy (hereafter referred to as autophagy) is a highly conserved and selective process that delivers cytosolic components to the lysosome for degradation via the intermediate of a double-membrane vesicle, the autophagosome [278]. The exact mechanism of cargo recognition remains obscure. However, the molecular characterization of autophagy receptors, initially SQSTM1/p62 and NBR1 (neighbor of BRCA1 gene 1) (Figure 1) has demonstrated that a Ub-dependent sensor system is involved in substrate specificity [279]. These autophagy receptors bind both to Ub and Atg8/LC3 (microtubule-associated protein 1 light chain 3) proteins, which, upon reversible conjugation to phosphatidylethanolamine, mediate the elongation and complete closure of the isolation membrane of the autophagophore. An active role of UbK63 chains in autophagy is supported by the properties of the UBDs of several of these autophagic receptors (p62, NBR1), the specificity of the E3 ligases involved, namely Parkin (Figure 1) and TRAF6, and, in some cases, the detection of this modification on cargoes. In addition, UbK63 modification of the autophagic proteins ULK1 [280] and Beclin 1 [281], both of which regulate autophagosome formation, activates autophagy. In the following section, we will discuss specific examples of how UbK63 polyubiquitylation is involved in autophagic removal of protein inclusions, mitochondria, and pathogens (Figure 3). Defective function of these autophagic pathways has been systematically linked to neurodegenerative processes, as detailed below.

UbK63 chains positively regulate the autophagic clearance of aggresomes, mitochondria and intracellular bacteria by interfering with various steps of the process. **A**. UbK63 chains on protein aggregates interact with distinct Ub-receptors to promote microtubule-dependent clustering at the MTOC (aggresome formation), autophagosome formation and fusion with the lysosome. DUBs played a role in two steps of the process. **B**. Mitochondria are marked by UbK63 chains to promote both perinuclear clustering and subsequent recruitment of the autophagic machinery. Targets and receptors of UbK63 chains on mitochondria remain ill-defined. **C**. Bacteria that escape the cytosol associated with remnant host membranes are rapidly decorated with UbK63 chains by host E3s to trigger autophagy via the simultaneous recruitment of multiple Ub-receptors.

### 5.1. The Aggresome-Autophagy Pathway

In the cell, aggresomes constitute the final line of defense against protein aggregates, when the (re)folding capacity of molecular chaperones and the proteolytic activity of 26S proteasomes are impaired or overwhelmed (reviewed in [282,283]). These pericentriolar structures are formed by dynein-dependent retrograde transport of protein aggregates to the microtubule-organizing center (MTOC), and they recruit the autophagic machinery for further clearance. The aggresome-autophagy pathway is critically involved in protection against neurodegeneration, and intraneuronal inclusion bodies show remarkable biochemical and morphological resemblance to aggresomes (reviewed in [284]).

UbK63 chains have been shown to decorate both protein aggregates in cell cultures [79,80,81,82,285,286] (see also Table 1) and intraneuronal inclusions of *post mortem* tissues from patients with neurodegenerative diseases [287]. It is reasonable to assume that this type of modification would serve as a signal that diverts aggregates from the proteasome under conditions in which its proteolytic activity is compromised. Consistent with this assertion, in cells treated with the proteasome inhibitor MG132, recruitment of Ubc13 by Parkin was dramatically and selectively enhanced, and Parkin-dependent K63-linked polyubiquitylation in the detergent-insoluble fraction was also increased [288]. However, several data suggest that UbK63 modification of aggregates constitutes more than a diversion signal and positively regulates both aggresome formation and autophagic clearance.

Consistent with a role for this type of modification in aggresome biogenesis, the occurrence of inclusion bodies and the targeting of specific aggregation-prone proteins into aggresomes was enhanced in response to increased UbK63 ubiquitylation [289]. In addition, K63-specific enzymes, namely Parkin, TRAF6 and the DUB Ataxin 3 (Figure 1 and Box 5), are positive regulators of aggresome formation and interact with their respective substrates not only at mature aggresomes but also at preaggresomal structures that accumulate when microtubule polymerization is inhibited [81,83]. To date, the microtubule-associated histone deacetylase HDAC6 constitutes the only functional link between aggresome formation and UbK63 ubiquitylation. HDAC6 binds both to dynactin and ubiquitylated proteins via its *C*-terminal Ub-binding zinc-finger domain (ZnF or BUZ, Figure 1), and recruits misfolded proteins to dynein motors for transport to aggresomes [290]. HDAC6 was shown to specifically interact with the UbK63 polyubiquitylated mutant isoform of DJ-1, a protein that is involved in familial Parkinson’s Disease. This finding suggested a role for this type of linkage in retrograde transport towards the MTOC [81]. However, a recent study investigating the structure of the HDAC6 BUZ domain revealed that it binds exclusively to the unanchored *C*-terminal diglycine motif of Ub and not to conjugated polyUb [291]. Interestingly, aggresomes were found to contain unanchored Ub chains, and their generation was dependent on the presence of Ataxin 3. Hence, the mechanism by which UbK63 chains promote aggresome maturation is far from being understood.

When UbK63 ubiquitylation is enhanced, the resulting Ub-positive inclusions show greater colocalization with the autophagy receptor p62 [286] (Figure 1 and Box 4) and are more amenable to autophagy [82,286,289] than those formed when UbK63 ubiquitylation is inhibited. In addition to p62, NBR1 (Figure 1 and Box 4) was identified as an autophagy receptor that is involved in the elimination of aggregated proteins [227]. NBR1 shares the same domain architecture as p62, and its UBA domain shows a slight preference for the UbK63 *versus* the UbK48 linkage *in vitro*. Finally, HDAC6 also promotes aggresome degradation, although it is not involved in the activation of autophagy, such as p62 or NBR. HDAC6 appears to interfere with later steps of the process, and, by remodeling the actin cytoskeleton, stimulates both fusion between autophagosomes and lysosomes and aggresome disaggregation [292,293]. Binding of HDAC6 to free UbK63 chains appears to be crucial for these functions. Surprisingly, Poh1, a DUB of the 19S particle, is involved in generating these unanchored chains, and injection of free UbK63 chains restores aggresome degradation in Poh1-deficient cells [292].

### 6.2. Mitophagy

Mitophagy is involved in organelle quality control, cellular metabolic adaptation and developmental programs such as maternal mitochondrial inheritance or erythrocyte maturation. Most data concerning the involvement of Ub in mitochondrial autophagic clearance were obtained from the study of the PINK1/Parkin quality control pathway (reviewed in [294]). Like Parkin, the mitochondrial Ser/Thr kinase PINK1 (PTEN-induced putative kinase 1) is involved in familial Parkinsonism, and their common pathway of mitochondrial quality surveillance is suspected to play a crucial role in neurodegeneration (reviewed in [295]). In this pathway, PINK1 constitutes the sensor of mitochondrial quality, as it accumulates at the surface of damaged mitochondria that fail to import proteins into the matrix. Mitochondrial accumulation of PINK1 triggers the recruitment and activation of cytosolic Parkin, which orchestrates, in a Ub-dependent manner, all of the subsequent steps of mitophagy: isolation of damaged organelles from the healthy mitochondrial network and perinuclear clustering, rupture of the outer mitochondrial membrane by proteasomal degradation, and targeting of remnant organelles to the lysosome by the autophagic machinery.

In cultured cells, activation of the PINK1/Parkin pathway is achieved by treatment with the uncoupler CCCP (carbonyl cyanide m-chlorophenyl hydrazone), which causes membrane potential collapse, mitochondrial import blockade and PINK1 accumulation. Proteomic analysis revealed that Ub is nine-fold more abundant in CCCP-treated than in healthy mitochondria [296]. More precisely, three types of linkages have been associated with the clearance of depolarized mitochondria: UbK48, UbK63 and K27-linked Ub (UbK27) chains [77,296,297,298,299].

UbK48 ubiquitylation correlates well with the recruitment of proteasomal subunits and proteolysis of outer mitochondrial membrane components in CCCP-treated mitochondria [296], a step that is limiting for further elimination by mitophagy [296]. However, UbK63 and UbK27 ubiquitylation are involved in the translocation of Parkin to mitochondria and the progression of mitophagy [77]. More precisely, UbK63 ubiquitylation of depolarized mitochondria has been linked to the mitochondrial targeting and catalytic activation of Parkin [77], p62-dependent juxtanuclear clustering of damaged organelles [298] and recruitment of the autophagic machinery *via* p62 or HDAC6 [77,298,300]. Although an exhaustive list of Parkin substrates following mitochondrial depolarization has been recently published [301], the type of ubiquitylation present on most of these substrates remains to be defined. To date, UbK63 chains only have been detected on Parkin itself [77] and on PINK1 [78] (Table 1).

A spatiotemporal regulation of UbK48 and UbK63 ubiquitylation also has been observed during autophagy of paternal mitochondria in the fertilized egg of *C. elegans* [302,303]. In both *Drosophila* and mice, UbK63 chains decorate sperm mitochondrial derivatives (MDs) soon after fertilization [304,305]. Ectopic maternal expression of yeast Ubp2 leads to a moderate yet significant delay in MD clearance compared to wild-type eggs in *Drosophila*, again underlying the involvement of UbK63 chains in efficient mitophagy [305].

### 6.3. Xenophagy

Intracellular bacteria often reside within membrane compartments of host cells. However, some pathogens escape into the cytosol and activate the host autophagic machinery in a process called xenophagy. Autophagic capture is often dependent on the presence of a Ub coat that is rapidly formed around bacteria and their associated membrane remnants after translocation to the cytosol [306,307].

The presence of UbK63 chains on the Ub coat of cytosolic bacteria has been demonstrated in the case of *Salmonella* [299] and *Mycobacterium* species [308,309,310]. UbK63 ubiquitylation of cytosolic *M. tuberculosis* was found to depend on Parkin, and Parkin-deficient mice and flies displayed increased susceptibility to various intracellular bacterial infections [309]. However, when challenged with *Salmonella*, Ubc13-deficient MEFs recruit LC3 to Ub-positive bacteria as efficiently as wild-type MEFs, suggesting that, in this case, xenophagy does not solely depend on UbK63 ubiquitylation [311]. Indeed, elimination of *Salmonella* by autophagy was found to be dependent on the RING E3 LRSAM1 (leucine-rich repeat and sterile alpha motif containing 1), which preferentially catalyzes the *in vitro* formation of K6-linked (UbK6) and UbK27 chains on bacteria. Other types of linkages associated with cytosolic bacteria include UbK48 chains detected on the Ub coat of *M. tuberculosis* [309,310] and linear chains on the Ub coat of *Salmonella* [299].

This diversity of Ub signals allows the recruitment of multiple autophagic receptors on cytosolic bacteria: p62 [312,313], NDP52 (nuclear domain 10 protein 52 [312,314], which contains a ZnF domain and binds to the bacterial glucan galectin-8 [315], and optineurin (OPTN) [316], which contains both a UBAN (Ub binding in ABIN and NEMO) and a ZnF domain. Interestingly, NDP52 and OPTN colocalize at the same subdomains of Ub-positive *Salmonella*, whereas p62 is found at distinct patches [312,316]. Silencing of either OPTN/NDP52 or NDP52/p62 had no additive effect on the increase in bacteria proliferation, which suggests that these receptors constitute non-redundant components of a unique pathway [312].

Regardless of the type of ubiquitylation, the identities of the ubiquitylated substrate(s) on cytosol-exposed bacteria remain elusive. Surprisingly, proteomic analysis of LRSAM1-modified substrates failed to identify any bacterial target [317]. In fact, it remains unknown whether ubiquitylation targets the bacteria themselves or the host proteins. Several ultrastructural studies have demonstrated that the Ub coat associates with remnant endosomal membranes on cytosolic bacteria [308,311,318,319,320]. The direct involvement of ubiquitylated host proteins in the induction of xenophagy has been recently proposed [311].

## 7. Concluding Remarks and Future Directions

More than 15 years ago, UbK63 chains were reported to modify a few plasma membrane proteins in yeast. Since then, their occurrence and potential roles at various steps of intracellular trafficking has increased exponentially. UbK63 chains of different lengths and conjugated by different E2/E3 complexes were shown to modify plasma membrane endocytic cargoes, MVB cargoes, viral proteins in the process of budding, or proteins to be degraded by various forms of autophagy. If the role of UbK63 as a specific internalization signal at the plasma membrane is limited to rare cases [32,35], there is a general consensus in diverse organisms that these chains act as a specific sorting signal for MVB targeting and subsequent lysosomal degradation [15,55,58,64,72], as well as a general strategy for sorting to the autophagic system [77,309]. Concerning the roles of UbK63 chains in pathology, a link is clear in the case of autophagy and neurological disorders [321]. Otherwise, if many E2/E3 or DUBs involved in assembly/disassembly of UbK63 are known to be mutated in a number of diseases, these enzymes have so many known or unknown targets that it is hazardous to conclude about a link between their mutations and impairment in precise trafficking functions.

Although structural data document why some of the involved E2s and/or E3s specifically promote the assembly of UbK63 chains on their substrate, this question remains open in many other cases. In fact, there is growing biological evidence that UbK63 chain formation depends not solely on the E2/E3 interaction but also on complexes between an E3 and a DUB (TRAF6 and CYLD [33], Nedd4-2- and USP2-45 [182]), or a substrate, its E3 and its Ub receptor (TrkA-TRAF6-p62 [322]). Whether these interactions or other mechanisms determine the length of the Ub chains added to the substrates remains to be determined. Interestingly, it was shown in an *in vitro* system that Ub chain elongation on an Rsp5 substrate is regulated by an interaction with a UBD-containing protein [323].

The mechanism(s) by which UbK63 chains on endocytic, MVB or autophagic cargoes interact with their binding partners at an atomic level remains to be elucidated. A host of biological assays involving UbK63 chains have clarified different pieces of a gigantic puzzle that has not yet been solved. This is most likely because biological evidence is growing much faster than the number of structures involving UbK63 chains. Nevertheless, based on our current knowledge and from a structural point of view, it seems that the preference/specificity of a given protein for UbK63 chains is related to a subtle cocktail comprising not only an adequate structure but also some dynamics/plasticity that allow an interaction with the latter chains. In an effort to obtain detailed information at an atomic level, structural biologists struggle in the study of multi-domain proteins, the only possible structures that account for the eventual cooperative effects induced by interactions with UbK63 chains. One example is the experimental results obtained for the interaction of the VHS-UIM construct with Lys-Ub_2_ chains [207]. Indeed, the individual domains VHS or UIM are not able to capture the cooperative effect that characterizes the interactions between VHS-UIM and Lys63-Ub_2_ and most likely explain the observed optimal efficiency of MVB cargo sorting [15,58].

UbK63 chains involved in trafficking do not act solely as sorting signals. They play a role in conformational changes (in the case of IFNAR-1 or the mitofusin Mfn2) [48,76], sometimes resulting in activation processes (for instance, WASH) [69]. They also aid in the formation of complexes by recruiting other proteins (such as the transient complexes formed in the IL1-dependent NFκB pathway) [276]. Similar functions can be anticipated in the study of the actin cytoskeleton, in which ubiquitylation/deubiquitylation events were observed [173,174]. These events may have involved UbK63 chains given the role of the E3 Rsp5 in modifying a number of cytoskeletal proteins at endocytic sites [148,151]. The effect of UbK63 ubiquitylation on the subcellular localization of paxillin [75], still poorly documented, may involve similar mechanisms.

The rapid progress in mass spectrometry (MS) (higher resolution, accuracy, sensitivity, speed) has revealed a global increase in UbK63-modified species, for instance, in animal models of neurodegenerative disease submitted to various treatments [324]. Progress in these techniques will allow the identification of novel proteins that are modified by UbK63 chains, even proteins that are present at low abundance. The combination of improved MS techniques and the use of UbK63-specific antibodies have already permitted the identification of novel UbK63 targets: for example, it was unexpected to observe the transient appearance of UbK63-modified E3s or transporters after EGF treatment of cultured cells [63]. The power of these antibodies in immunofluorescence studies has already helped to identify intracellular compartments that display proteins modified by UbK63 chains [58,63,199], and progress in this area is anticipated. The location of cellular compartments enriched in UbK63-modified proteins will also be documented using the recently developed GFP-tagged Ub sensors [299,325], which represent a powerful tool to track the dynamics of proteins that display this modification and to probe their function. The potential enrichment of proteins displaying such modifications by affinity purification using tandem-repeated–Ub-binding entities (TUBES) of appropriate specificity [326] will undoubtedly increase the efficiency of the identification of substrates carrying UbK63 chains. Such approaches should provide important insight into the process of autophagy, for which knowledge regarding the targets of UbK63 ubiquitylation is limited. The development of tools enabling the complete elimination of UbK63 chain formation, notably in human cells, will facilitate our understanding of the functions of this modification. All of these new tools will undoubtedly lead to the exciting discovery of new trafficking steps that involve UbK63 chains.

At the plasma membrane, MVB, or Golgi-to-endosome sorting, the sorting machinery involves multiple UBD-containing proteins, which are themselves ubiquitylated. The characterization of the nature of the modification of these UBD-containing proteins and the potential role it plays in the ultrastructural organization of the machinery in time and space constitutes a challenge for future investigations.

In addition to UbK63 conjugated to substrate, free UbK63 chains, which were initially described regarding their role in signaling events [327], are now also recognized to play a role in aggresome formation and polyUb chain clearance [291,292]; however, the underlying mechanisms remain to be defined. In addition to UbK63 chains, all of the other types of chains have also been described to be involved in trafficking. The rare documented examples include UbK11 chains in association with UbK63 chains, which are involved in the down-regulation of MHC I [36], UbK29 chains for lysosomal degradation in the Notch pathway [328], UbK33 chains for post-Golgi trafficking [329], and UbK27 or UbK6 chains together with UbK63 in mitophagy or xenophagy, respectively [77,317]. The world of mixed or branched Ub chains is also emerging. This new fields of investigations, still largely under-explored, add another layer of complexity, as these chains would present a particular structural organization, prone to be detected by the correct multi-UBD containing receptors [330].

## Supplementary Materials

Supplementary materials can be accessed at: http://www.mdpi.com/2073-4409/3/4/1027/s1.
